# One dataset, four meta-analyses: synthesising mean effects and heterogeneity within and among populations

**DOI:** 10.1242/bio.062872

**Published:** 2026-10-13

**Authors:** Haley Lacza, Ayumi Mizuno, Coralie Williams, Malgorzata Lagisz, Shinichi Nakagawa

**Affiliations:** ^1^ Collaboration for Open Science and Synthesis in Ecology and Evolution (COSSEE), Department of Biological Sciences, Faculty of Science, University of Alberta, Edmonton, AB T6G 2E9, Canada; ^2^ Evolution and Ecology Research Centre, School of Biological, Earth and Environmental Sciences, University of New South Wales, Sydney, NSW 2052, Australia; ^3^ School of Mathematics and Statistics, The University of New South Wales, Sydney, NSW 2052, Australia

**Keywords:** lnRR, lnVR, lnCVR, Yield variability, Heterogeneity, Location–scale models

## Abstract

Ecological syntheses (meta-analyses) usually ask ‘what is the average effect?’ But many questions also depend on whether outcomes become more or less variable and whether effects are predictable across contexts. We show how one dataset can support a workflow that synthesises both mean effects and within-group variability while also separating heterogeneity among study-level effects from residual heterogeneity among effect sizes within studies. Using an organic versus conventional crop-yield dataset, we analyse mean effects with the log response ratio (lnRR; Model 1) and within-group variability with the log variability ratio (lnVR) and log coefficient of variation ratio (lnCVR; Model 2), all calculated from the same summary statistics (means, s.d.s. and sample sizes). We then use multilevel location–scale meta-regression to test whether fertiliser type shifts expected lnRR and lnVR/lnCVR effects (Models 3 and 4) and whether residual and study-level heterogeneity differ between contexts. These heterogeneity components respectively inform consistency among represented comparisons and transferability to a new study. Analysing mean effects, within-group variability and context-dependent heterogeneity together can provide a more informative description of ecological responses than focusing on average effects alone.

## Teaser

**Summary:** This study shows how one meta-analytic dataset can reveal differences in mean effects, within-group variability and heterogeneity across ecological contexts.

## INTRODUCTION

Ecological effects vary. The same ecological process or intervention, for example, can produce different outcomes across individuals, populations, species, and environments, and this variation is often large enough to alter statistical inference and to limit generalisation across contexts (Levin, 1992; Lawton, 1999; Chamberlain et al., 2014). This means that ecology may not be well served by questions that focus only on ‘the average effect’. Two further questions are routinely important: ‘how variable are outcomes?’ and ‘how consistent are effects across contexts?’ (Duncan and Kefford, 2021; Spake et al., 2023). These questions are related, but they operate at two different levels under a meta-analytic context. Outcomes can vary more or less within a population (interindividual variability; e.g. a treatment may benefit some individuals but harm others, widening the spread even if the mean changes little), and effect sizes can vary more or less across studies or populations (between-study heterogeneity; e.g. the same intervention shows a strong effect in some contexts but a weak effect elsewhere). Both levels matter for ecological inference and practical application because the usefulness of evidence often depends on its generalisability and predictability. Here, generalisability and predictability refer to whether a phenomenon or effect is observed consistently across different conditions or only under specific conditions. Therefore, meta-analysis should target both the mean effect (central tendency or ‘location’) and the variation effect (dispersion or ‘scale’), rather than treating variation as a nuisance to be averaged away (Nakagawa et al., 2015, 2025a; Senior et al., 2020; Viechtbauer and López-López, 2022).

Most ecological meta-analyses, however, are still mean centric. A typical synthesis estimates an overall mean effect (e.g. using the log response ratio, lnRR, or the standardised mean difference, *d*) (Hedges et al., 1999; Lajeunesse, 2011, 2015). Variation enters mainly in two limited ways. First, within-study variation (s.d.s.) is usually treated as input for sampling variances and weights, rather than as a biological outcome that might itself change with treatments or environments (e.g. an intervention might stabilise outcomes by reducing among-individual differences or destabilise them by amplifying sensitivity). Second, variation among effect sizes is summarised as ‘heterogeneity’, reported as *I*^2^ or as a random-effects variance component (often written as *τ*^2^) (Higgins and Thompson, 2002; Higgins et al., 2003), and commonly assumed to be constant across studies (i.e. ‘homoscedasticity’; in contrast, non-constant variance across a continuous variable or among groups is termed ‘heteroscedasticity’). Measuring heterogeneity in a meta-analysis is informative, but simply reporting it does not show how predictable or generalisable the average effect is across contexts. What is often more important is understanding how both effect sizes and their variability differ across contexts. For example, if effect sizes are similar in some settings but highly variable in others, such as under different ecological conditions or study designs, then relying on an overall average effect may be a poor way to predict outcomes in a new context.

Two complementary method developments allow us to extract much more from the same dataset (i.e. evidence base). First, the ‘meta-analysis of variation’ uses dispersion-based effect sizes such as the log variability ratio (lnVR) and the log coefficient of variation ratio (lnCVR) to quantify changes in within-population dispersion (interindividual variability) between comparison groups (e.g. heteroscedasticity between the control and treatment groups) (Nakagawa et al., 2015; Senior et al., 2020) (Fig. 1A–C). A major advantage is practical: these effect sizes can usually be computed from the same summary statistics already collected for mean-based synthesis (means, s.d.s., and sample sizes) (Nakagawa et al., 2015; Senior et al., 2020). Second, ‘location–scale’ meta-regression extends standard meta-regression models by allowing the amount of among-effect-size variability to depend on moderators, thereby testing whether heterogeneity can differ between different moderator groups or contexts (i.e. heteroscedasticity among moderator levels) (Viechtbauer and López-López, 2022; Nakagawa et al., 2025a). Throughout this paper, we use ‘variability’ to mean within-study/within-population dispersion (targeted by lnVR/lnCVR) and ‘heterogeneity’ to mean dispersion among effect sizes beyond sampling error, including between-study and residual within-study components, which can be quantified for both mean effects (e.g. lnRR or *d*) and variability effects (lnVR/lnCVR) via variance components or moderators in meta-analyses and meta-regression analyses (Fig. 1D–G).

Here, we illustrate ‘one dataset, four meta-analyses’ using the organic versus conventional crop-yield dataset compiled by Ponisio et al. (2015). We begin with the standard mean-effect synthesis (lnRR) to summarise average yield differences (Model 1). We then analyse lnVR/lnCVR to address a different but equally ecological question: namely, whether organic systems differ from conventional systems in within-group yield variability (Model 2), a question highlighted in Knapp and van der Heijden (2018). Next, we move from description to explanation by fitting two types of meta-regression models: (i) location (mean) meta-regression models to test which moderators shift average effects and (ii) multilevel location–scale models to test whether residual heterogeneity changes systematically with those moderators (Models 3 and 4; in addition, we also show how to model study-level heterogeneity) (Viechtbauer and López-López, 2022; Nakagawa et al., 2025a; Blowes, 2026). Note that Model 3 applies the location–scale framework to mean effects (lnRR), whereas Model 4 applies the same framework to variability effects (lnVR/lnCVR). We conclude with practical guidance on effect-size choice and reporting. We explain how to clearly distinguish changes in within-group variability from variation in effect sizes among studies and why it is important to retain and report the basic summary statistics (means, s.d.s., and sample sizes) needed to synthesise both mean effects and variability effects. Importantly, our illustrative examples are implemented in R using metafor (Viechtbauer, 2010), brms (Bürkner, 2017) and glmmTMB (Brooks et al., 2017; Kristensen et al., 2026; Williams et al., 2026 preprint), accessible via an online tutorial at https://ayumi-495.github.io/one-dataset-four-meta-analyses/.

## META-ANALYSIS OF MEAN

### Why lnRR remains a useful starting point

Mean-effect meta-analysis is usually where ecological synthesis begins: ‘what is the average effect?’ For two-group comparisons, a common choice is the lnRR because it represents proportional change, is interpretable on a multiplicative scale, and is widely used across ecological meta-analyses (Hedges et al., 1999; Lajeunesse, 2011, 2015). For each comparison (treatment *T* versus control *C*), where *i* indexes the effect size for an individual treatment–control comparison, let 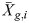 denote the sample mean, *s_g_*_,*i*_ the sample s.d., and *n_g_*_,*i*_ the sample size in group *g*∈{*T*, *C*}. The lnRR point estimate is:
(1)

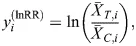
and its sampling variance is written as 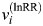. Under the standard assumption of independent groups, a common approximation is:
(2)

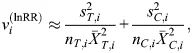
so each effect size enters the meta-analysis with its own known (approximated) plug-in precision (Hedges et al., 1999; Lajeunesse, 2010, 2015). We use *v_i_* throughout as shorthand for ‘the sampling variance of effect size *i*’, with the superscript indicating which effect size is being analysed (see also Senior et al., 2020, Nakagawa et al., 2023b).

**Fig. 1.**
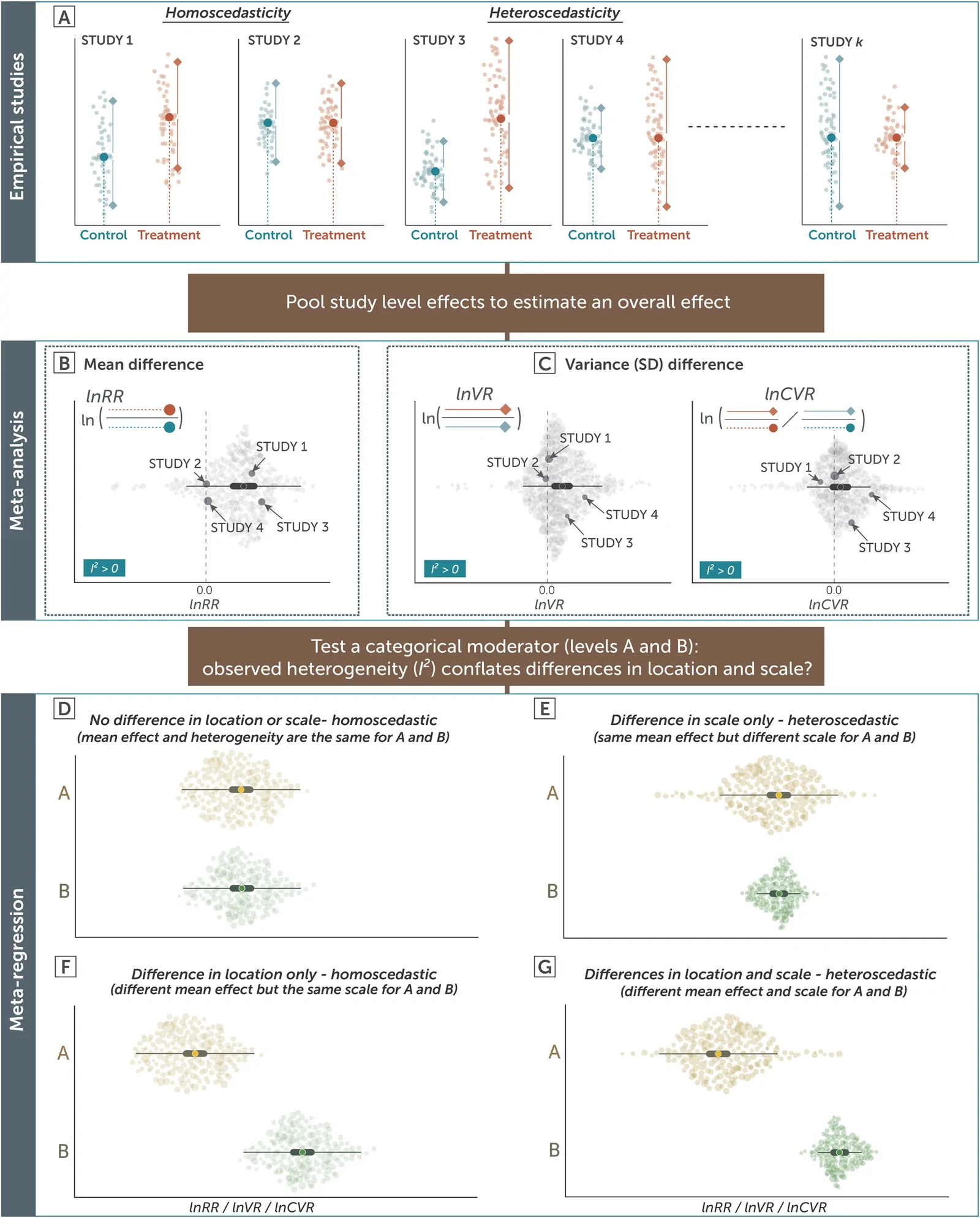
**Conceptual overview of synthesising mean effects, variability effects, and location–scale modelling of heterogeneity from empirical studies.** (A) Empirical two-group studies (control versus treatment) can be homoscedastic (similar within-group dispersion; Studies 1 and 2) or heteroscedastic (different within-group dispersion; Studies 3 and 4). Each study yields a study-level effect size that targets either a change in location (mean) and/or a change in scale (dispersion). (B) Mean effects are summarised with a mean-based effect size such as the lnRR, 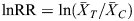, and then pooled across studies to estimate an overall mean effect (Model 1). (C) Within-population variability (dispersion) effects are summarised with dispersion-based effect sizes such as the lnVR (lnV R=ln(*s*_*T*_/*s*_*C*_)) and/or the lnCVR (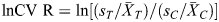) and then pooled across studies to estimate an overall variability effect (Model 2). In B and C, each point represents a study-level effect size; even when the pooled mean is near zero (vertical dashed line), effect sizes can vary substantially across studies, producing between-study heterogeneity (*I*^2^>0). (D–G) Location–scale meta-regression tests whether a categorical moderator shifts the expected effect (location) and/or residual heterogeneity among effect sizes within studies (scale; Models 3 and 4). The study-level extension additionally tests whether among-study heterogeneity differs between moderator levels and therefore informs transferability to a new study. (D) No difference in location or scale: the mean effect and heterogeneity are the same for A and B (homoscedastic). (E) Difference in scale only: mean effects are the same, but heterogeneity differs between A and B (heteroscedastic). (F) Difference in location only: mean effects differ between A and B, but heterogeneity is the same (homoscedastic). (G) Differences in location and scale: both mean effects and heterogeneity differ between A and B (heteroscedastic).

lnRR is a practical starting point because the ingredients needed to compute it (means, s.d.s., and sample sizes) are typically the same summary statistics required to compute lnVR and lnCVR. In other words, many existing datasets already contain what is needed to analyse mean effects and within-population variability side by side (Nakagawa et al., 2015; Senior et al., 2020). We do not focus on the standardised mean difference (*d*) in this article because ratio-scale outcomes are common in ecology (making lnRR a natural scale). In *d*, within-group s.d.s. are pooled and used to standardise the difference in means. Variation therefore enters primarily as a scaling quantity for the mean effect, rather than as an outcome whose difference between groups is estimated directly. Thus, although differences in within-group variability can influence the value of *d*, *d* does not directly quantify whether variability itself differs between groups. Our broader message here is to treat differences in variation themselves as targets of synthesis (Nakagawa et al., 2015; Senior et al., 2020). Importantly, the full workflow is most direct for two-group studies reporting means and s.d.s. because a mean-effect measure such as lnRR or the standardised mean difference can be paired with lnVR or lnCVR. Standardised mean differences use pooled within-group variation to scale a mean contrast but do not themselves estimate a between-group variability contrast. Odds ratios and correlations, commonly transformed to Fisher's z, can be fitted in multilevel location and heterogeneity models, including models in which residual or study-level heterogeneity varies with moderators. However, these measures alone do not provide the same mean-versus-variability decomposition without a separate dispersion-focused effect size.

### Multilevel meta-analysis as the default in ecology

A recurring issue in ecological meta-analysis is treating effect sizes as independent, recently highlighted by Peacor et al. (2025). In practice, multiple effect sizes commonly come from the same paper (e.g. different species, sites, years, outcomes, or treatment contrasts), and additional dependence can arise through shared controls or repeated measurements (Lajeunesse, 2011; Nakagawa and Santos, 2012). For this reason, a multilevel (random-effects) model is a sensible default, as it separates variation across different levels and avoids overstating precision (i.e. reducing inflated Type I error rates).

Following the notation used by Nakagawa and Santos (2012), a simple three-level model can be written as:
(3)

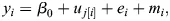

(4)

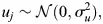

(5)

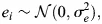

(6)

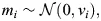
where *y_i_* is the observed effect size (here, 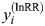), *β*_0_ is the intercept (overall or meta-analytic mean), *u_j_*_[*i*]_ captures between-study (or between-paper) differences, *e_i_* captures within-study differences among multiple effect sizes from the same study, and *m_i_* represents sampling error with known sampling variance *v_i_* (Lajeunesse, 2011; Nakagawa et al., 2025a). In practice, this model matches how ecological evidence is generated: individual studies (papers) often contribute clusters of related comparisons, and this dependence should be modelled rather than ignored. When sampling errors are correlated, for example, because multiple effect sizes share a control group, *m_i_* can be generalised to allow covariances. However, for notational simplicity, we retain the scalar form *v_i_* (assuming independence among sampling errors) rather than the full variance–covariance matrix representation (Williams et al., 2025).

This model is a multilevel random-effects meta-analysis, in which true effect sizes are allowed to vary across studies and comparisons rather than being assumed to share a single common value. This enables variance decomposition and makes explicit what is meant by ‘heterogeneity’ (variance not due to sampling error) and how it can be summarised. In simple random-effects meta-analyses (with a single study-level random effect in addition to sampling error), the total heterogeneity is often denoted by *τ*^2^. In the multilevel model considered here, this total heterogeneity is partitioned into a between-study component (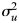) and a within-study component (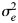), whose sum corresponds to *τ*^2^. A common descriptive summary of heterogeneity is *I*^2^, the proportion of the total variance in observed effect sizes that is attributable to heterogeneity rather than sampling error (Lajeunesse, 2011). For the multilevel model in Eqn 3, a convenient definition of the total heterogeneity, equivalent to standard *I*^2^ in the meta-analysis literature (Higgins and Thompson, 2002; Higgins et al., 2003), is:
(7)

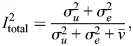
where 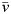 is a representative sampling variance (the mean or similar of *v_i_* across effect sizes) (Nakagawa and Santos, 2012). The same decomposition yields component-specific contributions,
(8)

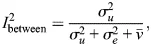

(9)

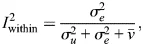
which indicate whether most heterogeneity sits between studies or among effect sizes within studies. These summaries are useful diagnostics, but they remain descriptive: because *I*^2^ is context dependent, large values (total or component specific) indicate that effects vary across contexts and motivate the next step, testing whether moderators explain heterogeneity in a meta-regression (Yang et al., 2025), which we demonstrate in the ‘Location–Scale Meta-Regression Models’.

### Example: organic versus conventional farming average yield

We used a subset of the organic versus conventional crop-yield database compiled by Ponisio et al. (2015); the dataset included 318 comparisons (effect sizes) for cereal crops (e.g. maize, wheat, barley, and oats), where organic systems were compared against conventional farming systems using standard synthetic inputs (control). Importantly, the type of fertiliser (animal versus plant derived) varied only within organic systems. We used this fertiliser-type variable as a moderator of the organic–conventional contrast, testing whether the magnitude of the organic–conventional effect varied with organic management. We fitted the three-level meta-analysis described above with study ID as a random effect (between-study effects) and effect (size) ID as another random effect (within-study effects); note that our model is for illustrative purposes, and, therefore, we do not claim that this is the best meta-analytic model (e.g. an alternative model could include another random effect for cereal types; for more details, see the online tutorial at https://ayumi-495.github.io/one-dataset-four-meta-analyses/). Our mean-effect synthesis based on lnRR answers a clear question (Fig. 1B): on average, how do organic and conventional yields compare? As with Ponisio et al. (2015), we found a clear decline in the average yield for organic farming by approximately 27% (Model 1 using metafor: *β*_0_=−0.32, 1−exp(−0.32)≈0.27, 95% confidence interval =[−0.43, −0.22]; Fig. 2A,C). Yet there was high heterogeneity (*I*_total_^2^=98.11%, *I*_between_^2^=46.09%, and *I*_within_^2^=52.02%; Fig. 2E), which warrants meta-regression analysis (see ‘Location–Scale Meta-Regression Models’; for comparable models using glmmTMB and brms, see the online tutorial at https://ayumi-495.github.io/one-dataset-four-meta-analyses/).

**Fig. 2.**
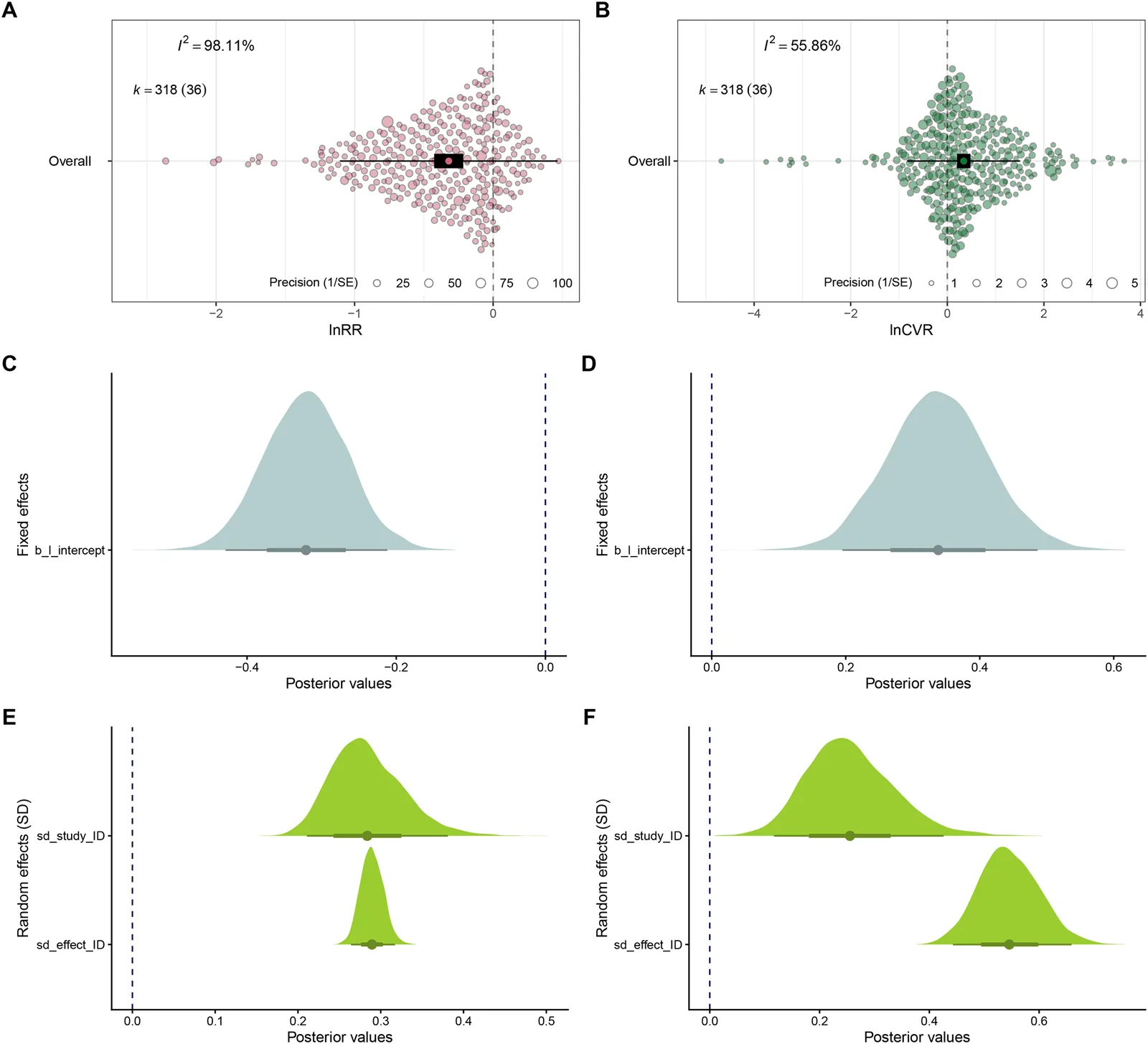
**Meta-analyses of mean yield differences and yield variability from a single evidence base.** A negative lnRR indicates lower organic yield, whereas a positive lnCVR indicates higher relative variability (within-group yield variability) under organic management. Panels A, C, and E summarise the meta-analysis of mean effects using the lnRR, and panels B, D, and F summarise the meta-analysis of within-group (mean-corrected) variability using the lnCVR, both comparing organic to conventional cereal yields (Ponisio dataset; *k* effect sizes with the number of studies in parentheses). (A,B) Orchard plots show individual effect sizes (points; size proportional to precision, 1/SE) with horizontal jitter to reduce overplotting. The black square denotes the pooled estimate from a multilevel random-effects model fitted using metafor; thick horizontal bars indicate the 95% interval for the pooled mean, and thin whiskers indicate the 95% prediction interval. The vertical dashed line marks zero (no organic–conventional difference). The reported *I*^2^ gives the proportion of total variation attributable to heterogeneity rather than sampling error. (C,D) Posterior distributions for the overall (intercept) effect in each model (*b*_0_=b_l_intercept), estimated using Bayesian multilevel models fitted in brms. (E,F) Posterior distributions for random-effects s.d.s. (study ID and effect-size ID). Points are posterior medians; thick and thin intervals denote 66% and 95% credible intervals, respectively. The R packages ggplot2 (Wickham, 2011), tidybayes (Kay, 2020) and orchaRd (Nakagawa et al., 2023a) were used to draw these plots.

## META-ANALYSIS OF VARIABILITY

### Why variability deserves its own meta-analysis

Mean effects describe shifts in central tendency, but many ecological questions are also about ‘spread’: do outcomes become more or less variable within populations when conditions change? Within-population variability is not simply nuisance variation. It can reflect differential sensitivity among individuals, temporal or spatial environmental variability, changes in trait distributions, or shifting demographic and community composition. Importantly, variability can change even when mean outcomes do not (Sánchez-Tójar et al., 2020; Senior et al., 2016a).

Despite this, standard meta-analytic workflows usually treat within-study s.d.s. mainly as inputs for sampling variances and weights (as in Eqn 2). As a result, a dataset can be used to make strong statements about mean effects while leaving within-group variability unanalysed. Meta-analysis of variability addresses this gap by analysing effect sizes that directly compare dispersion between groups, allowing the same evidence base to synthesise both mean outcomes and within-population variability (Nakagawa et al., 2015; Senior et al., 2020).

### Effect sizes for within-population variability: lnVR and lnCVR

For two-group designs (treatment *T* versus control *C*), within-population variability can be compared using two closely related dispersion-based effect sizes (Nakagawa et al., 2015; Senior et al., 2020). The lnVR targets differences in absolute dispersion (on the s.d. scale):
(10)

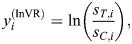
so 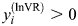 indicates higher within-population variability under treatment. lnVR is most natural when the scientific question is about absolute spread on the measurement scale (e.g. whether an intervention increases or decreases variability regardless of any shift in the mean). For independent groups, a commonly used large-sample approximation to the sampling variance is:
(11)

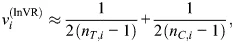
with small-sample bias corrections available and recommended when sample sizes are modest (Nakagawa et al., 2015; Senior et al., 2020).

The lnCVR targets dispersion relative to the mean:
(12)

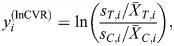
and is often preferred in ecology because s.d.s. frequently scale with means (including for yields, abundances, and performance measures) (Nakagawa et al., 2015; Senior et al., 2020). Put simply, lnVR asks whether the s.d. changes, whereas lnCVR asks whether the s.d. changes more (or less) than the mean. For independent groups, a convenient approximation to the sampling variance combines the lnVR component with the additional uncertainty from the means:
(13)

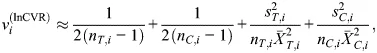
again with small-sample bias corrections and extensions for dependent designs (e.g. paired or pre-post data) given by Senior et al. (2020).

A major practical advantage is that both lnVR and lnCVR can usually be computed from the same summary statistics already extracted for lnRR (means, s.d.s., and sample sizes) (Nakagawa et al., 2015; Senior et al., 2020). Once calculated, they can be analysed using the same multilevel random-effects framework introduced for mean effects (Eqn 3), simply replacing 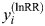 with 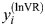 or 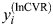 and using the corresponding known *v_i_*. The question shifts from mean effects to within-population variability, but the meta-analytic model remains the same (see Box 1).

Box 1. Meta-analyses of variability across fields: what lnVR and lnCVR add Meta-analyses usually synthesise differences in group means, but many applied and mechanistic questions hinge on whether an intervention (or condition) changes dispersion within groups: whether outcomes become more homogeneous or more heterogeneous. For example, the logic has been used to address questions that are naturally about heterogeneity among individuals. In nutrition/health, for example, variance-focused meta-analysis has been used to compare dietary interventions not only in terms of average weight-related outcomes but also in terms of whether they compress or spread the distribution of responses (i.e. whether benefits are broadly shared or concentrated in a subset) (Senior et al., 2016a).

In neuroscience/psychiatry, Brugger et al. (2020) used lnVR and lnCVR to test the hypothesis that schizophrenia is associated with increased interindividual variability in striatal dopamine function, meta-analysing dozens of studies and finding greater variability in dopamine receptor/transporter availability in patients than in controls. More broadly, the same ‘variance as an outcome’ framing has been applied to clinical efficacy questions, such as whether antipsychotic treatment effects differ in heterogeneity across patient groups, where the decision-relevant issue is not only the expected response but also how widely responses vary (Howes and Chapman, 2024).

In the social sciences, O'Dea et al. (2018) applied lnCVR at a very large scale (over 1.6 million students) to quantify differences in variability of academic grades between boys and girls, showing how dispersion meta-analysis can be used to test claims that are explicitly about variability rather than mean performance. Across these examples, the general idea is the same: synthesising lnVR/lnCVR alongside mean effects shifts the inferential target from ‘what happens on average’ to ‘how different do individuals respond’ and ‘how generalisable is the effect?’

These dispersion-based effect sizes cannot be calculated when within-group variability is not reported, such as when s.d.s. are missing or zero. In addition, you also cannot calculate lnCVR when group means are zero or nearly zero.

### Example: within-group yield variability under organic versus conventional farming

A meta-analysis of variability addresses a different ecological question: do organic systems differ in yield variability relative to conventional systems (Fig. 1C)? This question has been explicitly raised in the organic-yield literature, where lnCVR, along with lnVR, has been used to quantify relative yield variability and to connect synthesis to concerns about within-group yield variability (Knapp and van der Heijden, 2018). Using lnCVR is particularly natural here because yield variability often scales with mean yield; lnCVR therefore targets variability relative to mean yield (Knapp and van der Heijden, 2018). As with the previous meta-analysis, there was an increase in (within-group) relative variability in crop yield for organic farming by approximately 41% (Model 2 using metafor: *β*_0_=0.34, exp(0.34)−1≈0.41, 95% confidence interval, CI=[0.20, 0.48]; Fig. 2B,D). Also, we found moderate heterogeneity (metafor: 
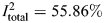
, 
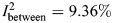
, and 
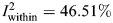
; Fig. 2F); this result indicated we require a further meta-regression analysis (see ‘Location–Scale Meta-Regression Models’; for comparable models using glmmTMB and brms, see the online tutorial at https://ayumi-495.github.io/one-dataset-four-meta-analyses/).

## LOCATION–SCALE META-REGRESSION MODELS

### From explaining average effects to explaining predictability

Meta-regression is usually motivated by the desire to explain why mean effects differ among studies: which ecological or methodological features shift the expected lnRR (yield gap) or lnCVR (within-group variability contrast)? However, in (applied) ecology, the more consequential question is often whether an effect is predictable in a new setting. Two management options can have similar mean effects yet differ sharply in how consistently those effects appear across contexts. Standard multilevel random-effects models acknowledge heterogeneity, but they typically assume that heterogeneity is constant across studies and moderator levels (homoscedasticity). Location–scale models relax this assumption by letting the ‘amount of heterogeneity’ itself depend on context, turning ‘it depends’ from a *post hoc* caveat into an estimable, testable component of the synthesis (Duncan and Kefford, 2021; Spake et al., 2023; Viechtbauer and López-López, 2022; Nakagawa et al., 2025a). Residual-scale moderation shows whether effects are more or less dispersed among comparisons within studies (Box 2). Box 3 extends the location–scale framework by giving separate scale sub-models for between-study and within-study heterogeneity. This distinguishes where context dependence occurs and whether variation among study-level effects changes with the moderator (Spake et al., 2022, 2023).

Box 2. Location–scale modelling as a general tool for discovering heteroscedasticity, not just meta-analysis A recurring limitation of ‘mean-only’ modelling is that it treats heteroscedasticity as an inconvenience: if residual variance differs among individuals, sites, years, taxa, or experimental settings, it is typically absorbed into a single error term and reported (at best) as overdispersion or ‘unexplained variability’ (Nakagawa et al., 2025a,b,c). Location–scale modelling flips this perspective by treating variability as an outcome in its own right. The conceptual move is simple: fit a model for the mean (location) in the usual way, and then fit a second model for the dispersion (scale), often by modelling 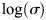 or 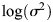 as a linear predictor with its own covariates. This allows the same predictors to be assessed for two distinct roles: whether they shift the expected response and whether they change predictability (the spread of responses around that expectation).

Because the scale component is interpretable, location–scale models directly address questions that are intrinsically about individual differences and context dependence. In behavioural ecology, for example, the ‘personality-predictability’ framing asks whether individuals differ not only in average behaviour but also in within-individual variability and whether those two components covary (Westneat et al., 2013; Cleasby et al., 2015; O'Dea et al., 2022). In quantitative genetics and comparative work, the same logic allows variance components (and their covariate dependence) to be treated as biologically meaningful traits, rather than as a single nuisance parameter (Mulder et al., 2008, 2016; Hill and Mulder, 2010; Sae-Lim et al., 2015; Nakagawa et al., 2025b). In primary research, this is often exactly what we care about: whether environmental stressors amplify interindividual differences, whether management interventions stabilise outcomes, whether phenotypes become more canalised in some contexts but more labile in others, or whether treatment effects are reliable enough to generalise.

Crucially, the location–scale idea is not limited to Gaussian-distributed data. Modern ‘distributional-regression’ implementations extend the same two-part thinking to many common outcome types by allowing separate sub-models for multiple distributional parameters, such as ‘shape’ sub-models (e.g. skewness and kurtosis) and zero- and one-inflated sub-models (Lee and Nelder, 1996, 2006; Rigby and Stasinopoulos, 2005; Stasinopoulos and Rigby, 2008). For proportions, dispersion (or overdispersion) can be modelled alongside the mean on binomial/beta-type scales; for counts, predictors can act on the mean rate while a separate component captures extra-Poisson variation (e.g. negative-binomial dispersion) and, where relevant, zero inflation (Nakagawa et al., 2025c). This matters in ecology because the empirical signature of ‘it depends’ is frequently the variance, not the mean: an ecological driver may have only a modest effect on the expected proportion or expected count yet strongly alter dispersion across sites, seasons, or taxa, signalling that the process is conditional on unmeasured context (cf. Duncan and Kefford, 2021; Spake et al., 2023).

Box 3. Extending location–scale models: where does heterogeneity occur? The location–scale model in Eqns 15–17 allows residual heterogeneity among effect sizes within studies to depend on moderators. In multilevel meta-analysis, however, heterogeneity can arise at more than one level. Studies can differ in their average effects (between-study heterogeneity), while effect sizes can also vary around those study-level averages (within-study heterogeneity). Extending the location–scale framework to both levels therefore allows us to ask not only whether heterogeneity changes with context but also ‘where that heterogeneity occurs’.

Using the notation introduced above, a general multilevel location–scale model can be written as:
(18)

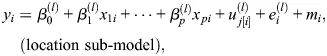

(19)

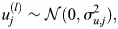

(20)

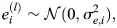

(21)

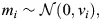
where *σ*_*u*,*j*_ represents between-study heterogeneity, *σ*_*e*,*i*_ represents residual within-study heterogeneity, and *v*_*i*_ is the known sampling variance. As in the model above, the sampling term can be generalised to a known variance–covariance matrix when sampling errors are correlated. Each source of heterogeneity can then be modelled with its own scale sub-model:
(22)

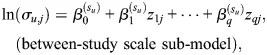

(23)

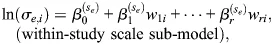
where the moderators in the three sub-models need not be the same (note that the between- and within-study moderators can often be different). The location coefficients, *β*^(*l*)^, describe how moderators shift the expected effect size; 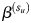 describes how moderators change heterogeneity among studies; and 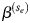 describes how moderators change residual heterogeneity among effect sizes within studies.

The scale coefficients are additive effects on the natural logarithm of an s.d. For a binary plant-versus-animal moderator, with animal-based fertiliser as the reference category, the corresponding contrasts on the natural s.d. scale are:
(24)

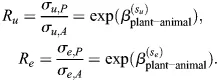

These s.d. ratios directly express the multiplicative difference in heterogeneity: a ratio of 1 indicates equal s.d.s., and a ratio above 1 indicates greater heterogeneity under plant-based fertilisers. When software models ln(*σ*^2^) rather than ln(*σ*), the corresponding s.d. ratio is 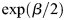.

This extension provides a more refined view of generalisability and transferability. Two contexts may have similar expected effects but differ in where heterogeneity arises. Greater between-study heterogeneity means that study-level effects vary more strongly around the expected effect, making that expectation less transferable to a new study. Greater within-study heterogeneity instead means that individual effect sizes are less consistent within studies, even if study-level averages remain similarly transferable. Therefore, a moderator can affect the location component, either scale component, or all three simultaneously.

Our organic–conventional yield example illustrates these different possibilities. For lnRR, between-study heterogeneity was greater under plant-based fertilisers than animal-based fertilisers (plant-to-animal s.d. ratio=2.18, 95% CrI=[1.11, 5.55]), whereas the difference in residual heterogeneity was uncertain (s.d. ratio=1.14, 95% CrI=[0.94, 1.40]). For lnCVR, the pattern was different: residual heterogeneity was greater under plant-based fertilisers (s.d. ratio=1.67, 95% CrI=[1.18, 2.34]), whereas the difference in between-study heterogeneity was highly uncertain (s.d. ratio=1.05, 95% CrI=[0.10, 8.63]). Thus, the same moderator can influence different levels of heterogeneity for different ecological outcomes.

Importantly, the extension preserved the broad finding that context dependence differed between lnRR and lnCVR, while showing that it arose at different levels. Estimating moderator effects on between-study heterogeneity nevertheless requires sufficient replication at the study level. In our example, only six studies contained both fertiliser categories, limiting information about category-specific study effects. We therefore treat this extension primarily as an illustration of how multilevel location–scale models can separate different sources of heterogeneity. Full model specifications, assumptions, sensitivity analyses, and prediction intervals for new studies are provided in the online tutorial at https://ayumi-495.github.io/one-dataset-four-meta-analyses/.

### Location–scale meta-regression: letting heterogeneity depend on context

A useful feature of location–scale modelling is that the same framework can be applied to different effect-size streams. In what follows, *y_i_* can represent a mean effect size (e.g. 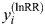) or a variability effect size (e.g. 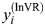
or 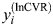). The sampling term *m_i_* is paired with the corresponding sampling variance *v_i_* for that effect size (e.g. 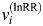, 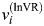, or 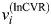). ‘Location’ therefore refers to the expected value of the chosen effect size, while ‘scale’ refers to the residual (within-study) heterogeneity in that effect size and how it changes with context. This allows moderators to be evaluated for two distinct roles: shifting the expected effect and altering residual dispersion among effect sizes within studies. Box 3 extends this framework to a third role: changing heterogeneity among study-level effects.

A multilevel location (mean) meta-regression can be written as:
(14)

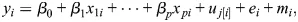
where *x*_1*i*_, …, *x*_*pi*_ are the values of *p* moderator variables for effect size *i* and *β*_*p*_ are their corresponding regression coefficients; the *β*s describe how moderators shift the average effect (whether the outcome is lnRR, lnVR, or lnCVR). This meta-regression model improves interpretation because it turns an overall average into a context-conditional average when a meta-analytic model (Eqn 3) finds non-zero heterogeneity, which is almost always the case for ecological datasets (Senior et al., 2016b).

Location–scale meta-regression extends Eqn 14 by allowing the residual (within-study) heterogeneity to vary with moderators. One convenient formulation is a double-hierarchical model in which the effect-size-level variance is modelled on the log scale:
(15)

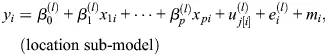

(16)

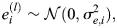

(17)

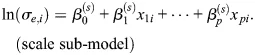


Here, the superscripts *l* and *s* denote the location (mean) and scale (variance or, more precisely, the natural logarithm of s.d.) components; note that metafor models, ln(*σ*^2^) whereas for brms and glmmTMB models, ln(*σ*). A non-zero residual-scale coefficient (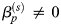) implies that dispersion among effect sizes within studies depends on the moderator: effects are more variable among the represented comparisons in some contexts than others (Viechtbauer and López-López, 2022; Nakagawa et al., 2025a; Blowes, 2026). This residual contrast should be distinguished from a moderator-dependent study s.d., which describes variation among study-level effects and is introduced in Box 3. Notably, Nakagawa et al. (2025a) introduce a complex location–scale model with the between-study effect on the scale part (i.e. 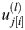), and an even more complex one with a correlation (*ρ*) modelled between 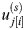 and 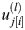; a positive correlation (*ρ*) indicates that, for example, larger effect sizes tend to have larger residual deviations (for these models, see the online tutorial at https://ayumi-495.github.io/one-dataset-four-meta-analyses/).

Importantly, a moderator in a location–scale model can matter in two qualitatively different ways. It can shift the average effect (captured in the location part), but it can also change the spread of effect sizes among comparisons within studies (captured in the scale part). The second case represents residual context dependence: the expected effect may not change much, but effect sizes can be more or less consistent among the represented comparisons (see Box 2). Box 3 extends this framework to ask whether heterogeneity among study-level effects also changes with the moderator.

### Example: organic fertiliser type moderates mean and variability effect

In the organic–conventional crop-yield dataset (Ponisio et al., 2015), organic fertiliser type (animal versus plant derived) is agronomically meaningful and plausibly linked to context dependence; this single moderator is used in both the location and scale components of the model to test shifts in the expected effect size and in residual heterogeneity (see Fig. 1D–G).

Specifically, we fitted multilevel location–scale meta-regression models with fertiliser type as the sole moderator and with random effects for study identity and effect-size identity to account for non-independence among comparisons. In the location and residual-scale sub-models, the animal-based intercept represents the expected effect and residual log s.d. for the reference category, and each plant contrast represents the corresponding shift (Fig. 3C,D).

**Fig. 3.**
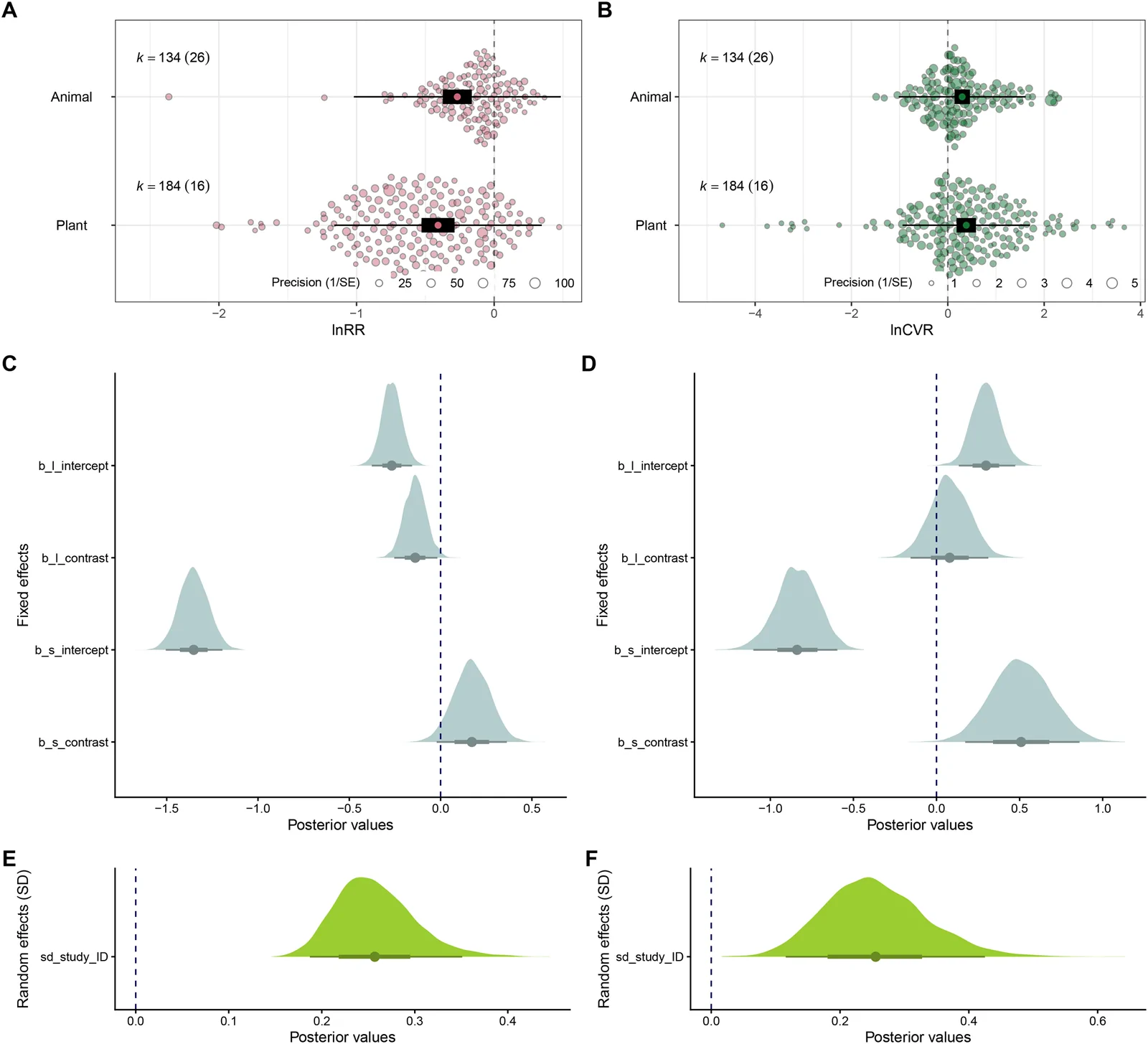
**Location–scale meta-regression showing how a moderator shifts expected effects and their residual heterogeneity.** Results are shown for lnRR (A,C,E) and lnCVR (B,D,F) when organic fertiliser type (animal versus plant derived) is used as a categorical moderator. *k* denotes the number of effect sizes (number of studies in parentheses). (A,B) Orchard plots stratified by fertiliser type. Points are individual effect sizes (bubble size ∝1/SE; horizontal jitter for visibility). Black squares and thick horizontal bars show the fitted subgroup mean and its 95% interval; thin whiskers show the 95% prediction interval. The vertical dashed line indicates no difference between organic and conventional farming. (C,D) Posterior distributions for fixed effects from a Bayesian location–scale model: location (mean) parameters (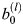 and 
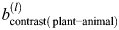
) and scale (heterogeneity) parameters (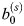 and 
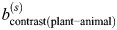
), where the scale coefficients act on 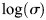 (positive values indicate greater residual heterogeneity). The intercept corresponds to animal-based fertiliser; the contrast gives the shift for plant-based fertiliser. (E,F) Posterior distributions for the between-study (random-effect) s.d. Points are posterior medians; thick and thin intervals denote 66% and 95% credible intervals, respectively. The R packages ggplot2 (Wickham, 2011), tidybayes (Kay, 2020) and orchaRd (Nakagawa et al., 2023a) were used to draw these plots.

Before turning to the results, we briefly remind the reader that Model 3 refers to a location–scale meta-regression of mean effects (lnRR), whereas Model 4 refers to an analogous location–scale meta-regression of variability (lnCVR).

For lnRR, the location part showed a negative average effect under animal-based fertilisers (Model 3 using brms: 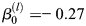, 95% credible interval, CrI=[−0.38, −0.16]), implying lower organic yields on average. The contrast coefficient (plant–animal) is also negative (
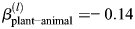
, 95% CrI=[−0.26, −0.03]), indicating an additional yield penalty under plant-based fertilisers relative to animal-based fertilisers. On the response-ratio scale, these correspond approximately to organic yields of 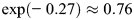 (about 24% lower than conventional) under animal-based fertilisers versus 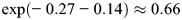 (about 34% lower) under plant-based fertilisers. The scale intercept for the animal-based reference category was 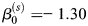 (95% CrI=[−1.45, −1.15]), corresponding to a residual s.d. of 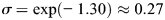 on the lnRR effect-size scale. This parameter describes residual heterogeneity in lnRR effect sizes around their expected value for animal-based systems; it is not a direct comparison of yield variability between organic and conventional farming. The scale contrast suggested a tendency towards greater residual heterogeneity under plant-based fertilisers (
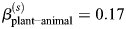
, 95% CrI=[−0.01, 0.35]), corresponding to about a 19% increase in residual heterogeneity relative to animal-based fertilisers (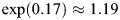), although the credible interval overlapped zero (Fig. 3A,C,E; see also Box 3).

For lnCVR, the location part indicated that organic systems tended to have higher mean-corrected within-group variability under animal-based fertilisers (Model 4 using brms: 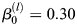, 95% CrI=[0.13, 0.47]; roughly 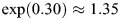, i.e. about 35% higher coefficient of variation in organic than conventional). The contrast (plant–animal) coefficient is small and highly uncertain (
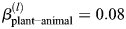
, 95% CrI=[−0.16, 0.31]), suggesting that fertiliser type did not strongly shift the average lnCVR contrast. In contrast, the scale part showed clear evidence that residual heterogeneity in lnCVR effects differed by fertiliser type (
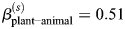
, 95% CrI=[0.17, 0.86]), implying a wider spread of lnCVR effects among comparisons within studies under plant-based fertilisers (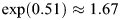; 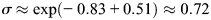). In other words, while the expected contrast in mean-corrected within-group yield variability is broadly similar between fertiliser categories, residual heterogeneity is greater under plant-based fertilisers, indicating a wider spread of lnCVR effects within studies (Fig. 3B,D,F; Box 3).

These paired results illustrate the useful extra insights which location–scale modelling could provide, and such insights are potentially decision relevant. Fertiliser type matters for lnRR largely through the expected yield gap (location), whereas for lnCVR, it matters mainly through the consistency of the within-group variability comparison (scale). Thus, a moderator can be important even when it barely shifts the mean effect because it may alter heterogeneity at one or more levels. Residual-scale moderation indicates whether effect sizes are more or less consistent among comparisons within studies, whereas moderator-dependent between-study heterogeneity, considered separately in Box 3, informs transferability to a new study (for comparable models using glmmTMB, see the online tutorial at https://ayumi-495.github.io/one-dataset-four-meta-analyses/).

### Conclusion and future directions

In this article, we make a simple claim: many ecological syntheses under-use the information already present in standard meta-analytic datasets. When primary studies report group means (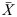), s.d.s (*s*) and sample sizes (*n*), the same evidence base can usually support inference about (i) average outcomes (via lnRR), (ii) within-group variability (via lnVR and lnCVR), and (iii) how both of these vary across contexts. The key is to treat ‘variability’ (dispersion within study groups or populations) and ‘heterogeneity’ (dispersion among effect sizes across studies) as distinct targets rather than a single catch-all notion of ‘variation’ (Senior et al., 2016b; Yang et al., 2025). Once that distinction is made, the analytical steps follow naturally: estimate mean effects, estimate variability effects, explain both with moderators, and then test whether predictability itself changes with context using location–scale models (Viechtbauer and López-López, 2022; Nakagawa et al., 2025a; Blowes, 2026).

The practical implication is that moderators can matter in two different ways. Some moderators primarily shift the expected effect (the ‘location’ component), changing what we should expect on average in a new setting. Other moderators primarily shift residual dispersion among effect sizes within studies, changing how consistent effects are among the represented comparisons. Transferability to a new study additionally depends on among-study heterogeneity. Multilevel location–scale modelling can estimate both components, and prediction intervals should propagate both (Box 3; cf. Spake et al., 2022). In the organic–conventional case study (Ponisio et al., 2015), placing lnRR and lnCVR side by side highlights not only the expected yield gap but also differences in within-group yield variability and whether those differences are consistent across agronomic contexts (Knapp and van der Heijden, 2018; Nakagawa et al., 2015; Senior et al., 2020). Location–scale modelling formalises that second question, replacing a generic ‘it depends’ with an explicit, testable statement about when effects are more or less heterogeneous (Viechtbauer and López-López, 2022; Nakagawa et al., 2025a; Blowes, 2026).

Three straightforward shifts would make this ‘one dataset, four analyses’ approach routine: one shift for all empiricists and two for meta-analysts. First, primary studies should consistently report the minimal summary statistics that enable both mean and variability synthesis (means, s.d.s., and sample sizes for each group), alongside design information that induces dependence (shared controls, repeated measures, multi-arm comparisons) (Lajeunesse, 2011; Nakagawa and Santos, 2012). Second, meta-analysts should treat mean-variance relationships as a modelling choice rather than a nuisance: lnVR is informative about absolute spread, whereas lnCVR targets variability relative to mean performance, and reporting both can clarify whether apparent changes in variability are driven mainly by shifts in the mean (Nakagawa et al., 2015; Senior et al., 2020). Third, applied syntheses should present results in prediction-oriented terms: pooled means and pooled variability effects are useful, but decision relevance often hinges on how widely effects vary across studies and whether moderators identify contexts where results are more transferable (Senior et al., 2016b; Nakagawa et al., 2021).

In short, the contribution here is not a new statistic but a suggested shift in a meta-analytic workflow in ecology: use the same dataset to estimate performance and within-group variability, and then use the same moderators to explain both the expected effects and their predictability (Nakagawa et al., 2015, 2025a; Senior et al., 2020; Viechtbauer and López-López, 2022). That workflow better matches what ecologists and decision-makers typically want to know, not only ‘what happens on average’, but also ‘how consistent is it’ and ‘how generalisable is this result across different contexts’ (Senior et al., 2016b; Yang et al., 2025; Spake et al., 2023). With this new workflow, let us make full use of the four meta-analytic models to better understand our changing world.

### Use of generative AI

Generative AI tools, including ChatGPT and Codex (OpenAI) and Claude Code (Anthropic), were used to assist with manuscript and tutorial drafting, code development, and consistency checks. All AI-assisted text and code were reviewed by the authors. These tools were not used as independent sources of scientific evidence. The authors take full responsibility for the manuscript, analyses and accompanying tutorial.

## Supplementary Material


