# Supplementary material for: One dataset, four meta-analyses: synthesising mean effects and heterogeneity within and among populations
Source: Biol Open. 2026 Oct 13;15(10):bio062872. doi: 10.1242/bio.062872 (PMC13714870; doi:10.1242/bio.062872)
Supplement: Supplementary file 1 [file biolopen-15-062872_review_history.pdf]

One dataset, four meta-analyses: synthesising mean effects and heterogeneity within and among populations

Haley Lacza, Ayumi Mizuno, Coralie Williams, Malgorzata Lagisz and Shinichi Nakagawa
10.1242/bio.062872

Editor: Daniel Gorelick

Review timeline

Original submission:	11 August 2026
Editorial decision:	19 August 2026
First revision received:	1 September 2026
Accepted:	1 September 2026

Original submission 1

First decision letter

MS ID#: bio.062872

MS Title: One dataset, four meta-analyses: synthesising mean effects and heterogeneity within and among populations

Authors: Haley Lacza; Ayumi Mizuno; Coralie Williams; Malgorzata Lagisz and Shinichi Nakagawa

I have read through both reviewers' comments. Both found the paper to be interesting and are enthusiastic about incorporating it into their work! The comments centred around the clarity of some of the wording and statements presented in the paper which seem relatively straightforward to adjust. I would encourage the authors to still fully address these critical points before acceptance. The reviewer reports are shown at the bottom of this email.

At this stage, we also ask you to ensure your manuscript complies with our formatting guidelines – please see our manuscript preparation guidelines for details. Provided you are able to fully address the referees' comments, we are positive about publication of your paper (we accept over 95% of revision submissions) and therefore hope you won't mind any extra work involved in reformatting your manuscript at this point.

Please upload both a 'clean' version of your Word file, along with a highlighted version clearly showing where you have made changes in the revised manuscript. Please avoid using 'Track changes' in Word files as these are lost in PDF conversion.

I should be grateful if you would also provide a point-by-point response detailing how you have dealt with the points raised by the reviewers in the 'Response to Reviewers' box. Please attend to all of the reviewers' comments. If you do not agree with any of their criticisms or suggestions please explain clearly why this is so.

Reviewer 1

Comments for the author

© 2026. Published by The Company of Biologists under the terms of the Creative Commons Attribution License (https://creativecommons.org/licenses/by/4.0/).

I very much enjoyed reading this manuscript, which addresses a number of issues that I believe are widespread in ecology, biology, environmental science, and (far) beyond. The manuscript is generally very well written and presented, conveys complex ideas as simply as possible while maintaining scientific accuracy, and has very clear and aesthetically appealing visualisations. I also believe that the paper is well suited to the journal and that, while the methods contained within the manuscript are not necessarily new, the paper is novel in bringing together these methods into a single four-part ecological meta-analytic workflow. Despite this, I think that several relatively minor issues need resolving before the paper is suitable for publication at Biology Open.

1. The model used in the paper allows the amount of residual variation among effect sizes within studies to change depending on the moderator. However, the manuscript sometimes describes this as showing that heterogeneity between studies changes and then uses this to make claims about how transferable (or generalisable) results are to other studies. Unless I'm misunderstanding something, these are not quite the same thing. As such, I feel that the authors should either extend the model so that study-level heterogeneity can also change with the moderator or revise the wording so that the conclusions match the level of variation that is actually being modelled. If transferability to a genuinely new study is a central claim of the project, the paper should also explain how both study-level variation and residual variation can contribute to uncertainty when making predictions.

2. The manuscript does not establish that the standard deviations in the underlying crop-yield dataset all measure the same kind of variation. For example, it is not clear whether they consistently represent variation across experimental replicates, variation across years, etc. Therefore, applying a variability effect size to these standard deviations does not automatically produce a measure of temporal yield stability. In my opinion, the safer interpretation would be that the analysis measures within-group yield variability. If the authors want to make claims specifically about temporal stability, they should demonstrate that the standard deviations used in the analysis consistently represent variation through time. Otherwise, the wording should probably be updated.

3. There are several small but sometimes actually important (when they affect the meaning) typesetting and grammatical issues throughout the manuscript. Please check carefully through the manuscript to ensure that these are corrected. I have tried to flag these below but I may have missed some.

Line-by-line comments:

* Page 2, line 20: 'Ecological syntheses (meta-analysis)' should probably be 'Ecological syntheses (meta-analyses)'.

* Page 3, lines 48-50: The sentence beginning 'Both levels matter for ecological inference and practical application...' is difficult to follow because the explanation of generalisation and predictability is inserted in the middle of the sentence. I suggest restructuring this so that the definition is given separately or in parentheses.

* Page 3, lines 60-62: There is a grammatical problem with 'non-constant variance across a continuous variable or among groups are called "heteroscedasticity"'. 'Variance' is singular, so this could instead read 'non-constant variance ... is termed heteroscedasticity'.

* Page 4, line 84: 'within population yield variability' should be hyphenated as 'within-population yield variability', consistent with its use elsewhere in the manuscript.

* Page 6, line 101: The Figure 1 caption states that the pooled mean effect is 'Model 2'. This should be Model 1, as Model 1 is the lnRR mean-effect analysis throughout the rest of the manuscript.

* Page 6, line 102: Throughout the manuscript, I suggest changing 'log variance ratio' to 'log variability ratio' for lnVR. As defined here, lnVR compares standard deviations rather than variances, so 'log variance ratio' is potentially misleading.

© 2026. Published by The Company of Biologists under the terms of the Creative Commons Attribution License (https://creativecommons.org/licenses/by/4.0/).

* Page 7, line 140: 'Using the notation adopted in the past work Nakagawa and Santos (2012)' is awkwardly phrased (it sounds like it is missing the word 'of'). I suggest something simpler, such as 'Following the notation used by Nakagawa and Santos (2012)...'.

* Page 8, line 168: 'We used a part of the organic versus conventional crop-yield database' would read more naturally as 'We used a subset of the organic versus conventional crop-yield database'.

* Page 8, line 173: 'three level meta-analysis' should be 'three-level meta-analysis'.

* Page 9, line 175: 'our model is for an illustrative purposes' should be 'our model is for illustrative purposes'.

* Page 9, lines 179-181: The reported I^2 values are presented as '98.11', '46.09', and '52.02', whereas I^2 is normally expressed as a percentage and Figure 2 reports the total I^2 this way. I suggest adding percentage signs here and checking that I^2 values are formatted consistently throughout the whole manuscript.

* Page 12, line 218: 'The question changes focus from mean to focus on within-population variability' repeats 'focus' and is a bit awkward. Maybe change to 'The question shifts from mean effects to within-population variability, but the meta-analytic model remains the same.'

* Page 12, lines 219-221: 'It would be worth mentioning that...' reads like an internal drafting note rather than final manuscript text?

* Page 15, lines 269-272: 'It is noted that Nakagawa et al. (2025a) introduces...' is a bit awkward. I suggest 'Nakagawa et al. (2025a) introduce more complex location-scale models...'.

* Page 21, line 357: Should be 'SN thanks Elena Gazzea'.

* Please check the capitalisation and punctuation of all CRediT contribution terms here for consistency, particularly 'Funding Acquisition', 'Project administration', and 'Writing - Original Draft'.

<u>Reviewer 2</u>

Comments for the author

Thank you for the opportunity to read this interesting work - it has changed my planned approach to an upcoming meta-analysis! Having read the work, I can confirm that it has proper controls and uses appropriate methods and tools. Appropriate statistical tests are used, and as noted by the authors, they do not claim that the modelling approach they use is the 'best' approach for their modelled dataset, rather that they illustrate a meta-analytic workflow. The sample size of the dataset they use to demonstrate this is satisfactory, and they use a dataset which has already been published elsewhere. In conduction with the linked online tutorial, the manuscript does provide sufficient detail for reproducibility, though I do have a number of comments on the tutorial (see below) to ensure it is easy to follow. The authors conclusions are supported by the data, and I cannot detect any flaws in the approach taken, though some additional information and small edits are suggested below.

The only major addition I would suggest making to improve the scholarship of the manuscript is to add more information / discussion about whether this approach can be applied to meta-analyses using other measures. Standardised mean difference is briefly referred to, but this could be expanded and made more explicit. It would also be good to explicitly state whether this approach can or cannot be applied to other measures, such as odds rations or associations (e.g. r or Fisher's z), and then discuss the implications of this. Other than this, I only have a few small suggestions, detailed below.

Line 47: I would suggest changing 'studies' to 'contexts' as it is really the differences between contexts which would be of interest to the ecologist, rather than the study per se.

© 2026. Published by The Company of Biologists under the terms of the Creative Commons Attribution License (https://creativecommons.org/licenses/by/4.0/).

Line 47-50: It may be as this is a long sentence, but I don't find it's meaning clear. Perhaps split into two sentences, or with 'whether a phenomenon or effect is observed consistently across different conditions or only under specific conditions,' in paratheses?

Line 64: I would change this to 'effect sizes and variance'. Because the rest of the paragraph seems to be discussion shortcomings in the treatment of variance, and the proposed framework focuses on an approach which can assess both.

Line 71-73: Add (Nakagawa et al., 2015; Senior et al., 2020) to support this statement.

I really like Figure 1, it does a great job of graphically demonstrating the main points of the introductory paragraph. Both E and G are referred to as homoscedastic heterogeneity, and both F and H as heteroscedastic heterogeneity. It would be easier to talk about these differences if there were different terms to describe each of these graphs.

Section 2 is very nicely described. One possible addition is on / around lines 129-130. As this paper may be read by those who are less familiar with all aspects of meta-analysis, it would be helpful to explicitly state why analysis using standardised mean difference (d) treats variation as a background assumption.

Legend for figure 2. I would move the text 'where negative lnRR implies lower organic yield and positive lnCVR implies higher relative variability (lower stability) under organic management' much earlier in the figure legend, perhaps in the 'title' of the legend.

Section 3 is also very nicely described and easy to follow. I have just a couple of small suggestions to ease readability. On line 191: perhaps 'a dataset' rather than 'the same dataset', as you are describing a single use of a dataset in this sentence.

On line 9 in box 1: As the text before and after the comma are complete, related thoughts, replace the comma with semi-colon, to read: '…within groups; whether outcomes…'

Lines 290-299: The model results (tutorial and figure 3) seems to suggest that organic systems using animal-based fertilizers are less variable (more predictable) than conventional farming - the estimate is -1.3. However, this is not mentioned in either the manuscript or the tutorial. I appreciate that the focus of the manuscript is the method rather than the specific results, but this seems like an omission and the interpretation of this number should be made explicit. Alternately, if I have misunderstood the interpretation of this -1.3, this would suggest that greater explanation for the reader is needed.

In figure 3 A and B, I would suggest switching the order of 'plant' and 'animal', so that 'animal' is first and the order matches that in graphs C and D (of 'intercept' then 'contrast'). In C and D, I would suggest switching the order of l and s (currently s then l, change to having l at the top of the graph) so it matches the order in both the text and the figure legend.

Line 315: I think this should be metafor and glmmTMB (as the models presented were done in brms), though I would argue that the models in metafor are not comparable, so I would suggest rewording to: 'for comparable models using glmmTMB, see the online tutorial'.

Online tutorial

I would suggest the following changes to ensure this is easy to follow.

1) Add a direct link to the dataset, and explicitly instruct the user to download this and load it so the code can be used unmodified.

2) In model 1, brms, meta-analysis (overall effect), a paragraph is repeated twice: 'One important point concerns how random effects are reported. In brms, random-effect estimates are presented as standard deviations (SD), whereas metafor reports variances. Therefore, to compare estimates between the two packages, the standard deviations reported by brms must be squared to obtain the corresponding variance estimates.'

© 2026. Published by The Company of Biologists under the terms of the Creative Commons Attribution License (https://creativecommons.org/licenses/by/4.0/).

3) Use of the function brm in brms requires a version of Rtools which is compatible with the R version being used. It may be helpful to users to add a note about this, as most other parts of the code (see exception below) run without any edits / additions needed.

4) In the glmmTMB section, there is a typo, in 'For meta-analysis, this grouping dactor should always have one level', correct to 'factor'.

5) When I run the model 2 InCVR section for both metafor and glmmTMB, I get very slightly different results to those in the tutorial e.g. for the metafor overall effect my estimate is 0.3397 and the I2_Total is 55.863504. I'm not sure what is causing this, as my results for model 1 and lnVR are identical. I also get a slightly different result to that reported in the tutorial for models mod_lnCVR_ls, mod_lnCVR_mr2, glmm_lnRR1 and glmm_lnCVR1. Model glmm_lnRR2 gives the error message: Error in fitTMB(TMBStruc) : negative log-likelihood is NaN at starting parameter values

6) In model 3-4 section 'It is best to treat the metafor models presented above as approximations' should read 'below'.

Reviewer's Responses to Questions

Experimental quality
Are experiments performed using appropriate methods that will answer the question (or test the hypothesis or support the observations) posed by the authors? Is the right tool used for the job?
If 'No', please indicate reasons in Comments for Author box below.
Reviewer #1:
- Yes

Reviewer #2:
- Yes

Were the data analyzed using appropriate statistical tests?
If 'No', please indicate reasons in Comments for Author box below.
Reviewer #1:
- Yes

Reviewer #2:
- Yes

Reproducibility
Were experiments performed using adequate number of biological replicates?
If 'No', please indicate reasons in Comments for Author box below.
Reviewer #1:
- Yes

Reviewer #2:
- Yes

Is there sufficient raw data to assess the rigor of the analysis?
If 'No', please indicate reasons in Comments for Author box below.
Reviewer #1:
- Yes

Reviewer #2:
- Yes

Does the methods section provide sufficient detail to permit reproducibility?
If 'No', please indicate reasons in Comments for Author box below.

© 2026. Published by The Company of Biologists under the terms of the Creative Commons Attribution License (https://creativecommons.org/licenses/by/4.0/).

Reviewer #1:

- Yes

Reviewer #2:

- Yes

Completeness
Are the manuscript's conclusions supported by the data?
If 'No', please indicate reasons in Comments for Author box below.
Reviewer #1:

- Yes

Reviewer #2:

- Yes

Scholarship
Do the authors cite and discuss the merits of data that would argue for and against their conclusion?
If 'No', please indicate reasons in Comments for Author box below.
Reviewer #1:

- Yes

Reviewer #2:

- Yes

Does the manuscript title & abstract accurately reflect the contents of the manuscript, without hyperbole?
If 'No', please indicate reasons in Comments for Author box below.
Reviewer #1:

- Yes

Reviewer #2:

- Yes

First revision

Author response to reviewers' comments

Comments from the Reviewers:

Reviewer 1

General feedback:

I very much enjoyed reading this manuscript, which addresses a number of issues that I believe are widespread in ecology, biology, environmental science, and (far) beyond. The manuscript is generally very well written and presented, conveys complex ideas as simply as possible while maintaining scientific accuracy, and has very clear and aesthetically appealing visualisations. I also believe that the paper is well suited to the journal and that, while the methods contained within the manuscript are not necessarily new, the paper is novel in bringing together these methods into a single four-part ecological meta-analytic workflow. Despite this, I think that several relatively minor issues need resolving before the paper is suitable for publication at Biology Open.
[R1] We thank the reviewer for their positive and encouraging assessment of our manuscript. We greatly appreciate their thoughtful comments and suggestions, which have helped us improve the manuscript's clarity and presentation.

© 2026. Published by The Company of Biologists under the terms of the Creative Commons Attribution License (https://creativecommons.org/licenses/by/4.0/).

1. The model used in the paper allows the amount of residual variation among effect sizes within studies to change depending on the moderator. However, the manuscript sometimes describes this as showing that heterogeneity between studies changes and then uses this to make claims about how transferable (or generalisable) results are to other studies. Unless I'm misunderstanding something, these are not quite the same thing. As such, I feel that the authors should either extend the model so that study-level heterogeneity can also change with the moderator or revise the wording so that the conclusions match the level of variation that is actually being modelled. If transferability to a genuinely new study is a central claim of the project, the paper should also explain how both study-level variation and residual variation can contribute to uncertainty when making predictions.

[R2] We thank the reviewer for identifying this important distinction. They are correct that the scale sub-model in our original analysis allowed residual heterogeneity among effect sizes within studies to vary with fertiliser type, while retaining a common among-study standard deviation. We have made two main changes.

First, we revised the manuscript throughout to distinguish residual heterogeneity among effect sizes within studies from heterogeneity among study-level effects. We also revised our definition of heterogeneity in the Introduction to explicitly encompass both between-study and residual within-study components [L72-75]. Residual-scale moderation is now interpreted in terms of the consistency of effect sizes among represented comparisons within studies, whereas transferability to a new study is linked specifically to between-study heterogeneity. Second, we added Box 3 and a fully reproducible tutorial extension showing how the location, between-study scale, and within-study scale components can be modelled separately for both lnRR and lnCVR. We present this extension in Box 3 because the full model described there cannot be fitted directly with the glmmTMB, brms, or metafor implementations used elsewhere in the tutorial.

Fertiliser category varies within some studies, and only six studies contained both animal- and plant-based fertiliser categories. We used independent animal- and plant-specific study effects in the primary full-data analysis and explicitly describe the limited information available for estimating category-specific between-study heterogeneity. The tutorial additionally presents a correlated parameterisation as an exploratory analysis, together with sensitivity analyses to clarify the consequences of this modelling choice. We report plant-to-animal SD ratios because these are the exponentiated scale contrasts on the natural SD scale and directly express the multiplicative difference in heterogeneity between fertiliser categories.

The extended analysis showed that the same moderator can be associated with heterogeneity at different levels for different outcomes. For lnRR, between-study heterogeneity was greater under plant-based fertilisers, with a plant-to-animal study-SD ratio of 2.18 (95% CrI = [1.11, 5.55]), whereas the residual-SD ratio was uncertain (1.14, 95% CrI = [0.94, 1.40]). Thus, for lnRR, moderator-dependent heterogeneity was evident primarily at the between-study level, implying lower transferability of the expected study-level effect under plant-based fertilisers, although the interval was relatively wide. For lnCVR, residual heterogeneity was greater under plant-based fertilisers (residual-SD ratio = 1.67, 95% CrI = [1.18, 2.34]), whereas the between-study difference was highly uncertain (study-SD ratio = 1.05, 95% CrI = [0.10, 8.63]). We interpret the lnCVR result as lower consistency among effect sizes within studies and do not claim that transferability of the study-level mean differs between fertiliser categories. The tutorial also demonstrates how both between-study and within-study heterogeneity contribute to prediction intervals for a new study.

We made corresponding changes in several places in the manuscript, including Sections 4.1-4.3 and the Conclusion, and added Box 3 to make these distinctions explicit.

Original:
We then extend standard meta-regression to location-scale (mean-variance) modelling, allowing moderators to explain not only how lnRR (Model 3) and lnVR/lnCVR (Model 4) shift on average (``location") but also how their within-study/residual heterogeneity changes with context (``scale"), thereby distinguishing settings where effects are generalisable

© 2026. Published by The Company of Biologists under the terms of the Creative Commons Attribution License (https://creativecommons.org/licenses/by/4.0/).

and transferable from those where they are strongly context-dependent.

Revised [L24-28]:
We then use multilevel location-scale meta-regression to test whether fertiliser type shifts expected lnRR and lnVR/lnCVR effects (Models 3 and 4) and whether residual and study-level heterogeneity differ between contexts. These heterogeneity components respectively inform consistency among represented comparisons and transferability to a new study.

Original:
Next, we move from description to explanation by fitting two types of meta-regression models: (i) location (mean) meta-regression models to test which moderators shift average effects, and (ii) location-scale (mean-variance) models to test whether the amount of between-study heterogeneity changes systematically with those moderators (Models 3 \& 4)

Revised [L80-84]:
Next, we move from description to explanation by fitting two types of meta-regression models: (i) location (mean) meta-regression models to test which moderators shift average effects, and (ii) multilevel location-scale models to test whether residual heterogeneity changes systematically with those moderators (Models 3 \& 4; in addition, we also show how to model study-level heterogeneity; Box 3)

Original:
Location--scale (mean--variance) meta-regression tests whether a categorical moderator (levels A and B) shifts the expected effect (location) and/or the predictability/transferability of effects (scale; heterogeneity) (Models 3 and 4).

Revised - Figure 1 caption:
Location-scale meta-regression tests whether a categorical moderator shifts the expected effect (location) and/or residual heterogeneity among effect sizes within studies (scale; Models 3 and 4). The study-level extension in Box 3 additionally tests whether among-study heterogeneity differs between moderator levels and therefore informs transferability to a new study.

Original:
This is the key step linking synthesis to generalisability and transferability: it tells us not only what to expect on average, but also in which contexts that expectation is reliable enough to apply to a new site or study

Revised [L232-236]:
Residual-scale moderation shows whether effects are more or less dispersed among comparisons within studies. Box 3 extends the location-scale framework by giving between-study and within-study heterogeneity separate scale sub-models. This distinguishes where context dependence occurs and whether variation among study-level effects changes with the moderator

Original:
This allows moderators to be evaluated for two distinct roles: shifting the expected effect and altering its predictability (uncertainty around the average) across studies.

Revised [L242-245]:
This allows moderators to be evaluated for two distinct roles: shifting the expected effect and altering residual dispersion among effect sizes within studies. Box 3 below extends this framework to a third role: changing heterogeneity among study-level effects.

Original:
A non-zero scale coefficient ($\beta_p^{(s)} \neq 0$) implies that the amount of heterogeneity depends on the moderator: effects are more variable in some contexts than others, so generalisation is correspondingly easier or harder (Viechtbauer and López-López, 2022; Nakagawa et al., 2025a; Blowes, 2026).

© 2026. Published by The Company of Biologists under the terms of the Creative Commons Attribution License (https://creativecommons.org/licenses/by/4.0/).

Revised [L257-261]:
A non-zero residual-scale coefficient ($\beta_p^{(s)}\neq 0$) implies that dispersion among effect sizes within studies depends on the moderator: effects are more variable among the represented comparisons in some contexts than others (Viechtbauer and López-López, 2022; Nakagawa et al., 2025a; Blowes, 2026). This residual contrast should be distinguished from a moderator-dependent study SD, which describes variation among study-level effects and is introduced in Box 3.

Original:
Importantly, a moderator in a location-scale model can matter in two qualitatively different ways. It can shift the average effect (captured in the location part), but it can also change the spread of effects across studies (captured in the scale part). The second case is often what readers mean by ``context dependence'': the expected effect may not change much, but the evidence becomes more or less predictable (see Box 2). This very point is well illustrated in the following example.

Revised [L265-270]:
Importantly, a moderator in a location-scale model can matter in two qualitatively different ways. It can shift the average effect (captured in the location part), but it can also change the spread of effect sizes among comparisons within studies (captured in the scale part). The second case represents residual context dependence: the expected effect may not change much, but effect sizes can be more or less consistent among the represented comparisons (see Box 2). Box 3 extends this framework to ask whether heterogeneity among study-level effects also changes with the moderator.

Original:
In these models, the reference level is animal-based fertiliser, so the intercept represents the expected effect (and heterogeneity) for animal-based systems, and the \texttt{contrast} coefficient represents the shift for plant-based systems

Revised [L279-280]:
In the location and residual-scale sub-models, the animal-based intercept represents the expected effect and residual log-SD for the reference category, and each plant contrast represents the corresponding shift (Fig. 3C-D)

Original:
In contrast, the scale part shows clear evidence that fertiliser type governs predictability (context dependence) of lnCVR effects ($\beta^{(l)}_{\texttt{animal-plant}}$ =0.52, 95\% CrI = [0.18, 0.86]), implying a much wider spread of lnCVR effects across studies for the plant-based fertilisers ($\exp(0.52)\approx1.68$; $\sigma\approx\exp(-0.83+0.52)\approx0.73$). In other words, while the expected stability gap (lnCVR) is broadly similar between fertiliser categories, the evidence is far less transferable under plant-based fertilisers: some studies show near parity in stability, whereas others show much larger organic-conventional differences (Fig.~\ref{fig:meta-regression}B,D,F)

Revised [L300-305]:
In contrast, the scale part showed clear evidence that residual heterogeneity in lnCVR effects differed by fertiliser type ($\beta^{(\rev{s})}_{\rev{\texttt{plant-animal}}}$ =0.51, 95\% CrI = [0.17, 0.86]), implying a wider spread of lnCVR effects among comparisons within studies under plant-based fertilisers ($\exp(0.51)\approx1.67$; $\sigma\approx\exp(-0.83+0.51)\approx0.72$). In other words, while the expected contrast in mean-corrected within-group yield variability is broadly similar between fertiliser categories, residual heterogeneity is greater under plant-based fertilisers, indicating a wider spread of lnCVR effects within studies (Fig. 3B,D,F; Box 3).

Original:
Thus, a moderator can be important even when it barely shifts the mean effect: it can determine whether an estimated effect is robust and transferable, or whether it is strongly contingent on unmeasured ecological and methodological details within that moderator

© 2026. Published by The Company of Biologists under the terms of the Creative Commons Attribution License (https://creativecommons.org/licenses/by/4.0/).

category (for comparable models using metafor and brms, see the online tutorial: here is the latest online tutorial - https://ayumi-495.github.io/one-dataset-four-meta-analyses/).

Revised [L309-313]:
Thus, a moderator can be important even when it barely shifts the mean effect because it may alter heterogeneity at one or more levels. Residual-scale moderation indicates whether effect sizes are more or less consistent among comparisons within studies, whereas moderator-dependent between-study heterogeneity, considered separately in Box 3, informs transferability to a new study (for comparable models using glmmTMB, see the online tutorial).

Original:
Location-scale meta-regression showing how a moderator shifts expected effects and their residual predictability

Revised [Figure 3 title]:
Location-scale meta-regression showing how a moderator shifts expected effects and their residual heterogeneity

Original:
Other moderators primarily shift the predictability of the evidence (the ``scale" component), changing how transferable (generalisable) that expectation is (cf., Spake et al., 2022).

Revised [L327-331]
Other moderators primarily shift residual dispersion among effect sizes within studies, changing how consistent effects are among the represented comparisons. Transferability to a new study additionally depends on among-study heterogeneity. Multilevel location-scale modelling can estimate both components, and prediction intervals should propagate both (Box 3; cf., Spake et al., 2022).

Added: Box 3 (L314-315) - see below.

© 2026. Published by The Company of Biologists under the terms of the Creative Commons Attribution License (https://creativecommons.org/licenses/by/4.0/).

Box 3 — Extending location–scale models: Where does heterogeneity occur?

The location–scale model in Equations (15)–(17) allows residual heterogeneity among effect sizes within studies to depend on moderators. In multilevel meta-analysis, however, heterogeneity can arise at more than one level. Studies can differ in their average effects (between-study heterogeneity), while effect sizes can also vary around those study-level averages (within-study heterogeneity). Extending the location–scale framework to both levels therefore allows us to ask not only whether heterogeneity changes with context, but also "where that heterogeneity occurs?".

Using the notation introduced above, a general multilevel location–scale model can be written as

$$y_i = \beta_0^{(l)} + \beta_1^{(l)} x_{1i} + \cdots + \beta_p^{(l)} x_{pi} + u_{j[i]}^{(l)} + e_i^{(l)} + m_i, \qquad \text{(location sub-model)} \tag{18}$$

$$u_j^{(l)} \sim \mathcal{N}(0, \sigma_{u,j}^2), \tag{19}$$

$$e_i^{(l)} \sim \mathcal{N}(0, \sigma_{e,i}^2), \tag{20}$$

$$m_i \sim \mathcal{N}(0, v_i), \tag{21}$$

where $\sigma_{u,j}$ represents between-study heterogeneity, $\sigma_{e,i}$ represents residual within-study heterogeneity, and v_i is the known sampling variance. As in the model above, the sampling term can be generalised to a known variance–covariance matrix when sampling errors are correlated. Each source of heterogeneity can then be modelled with its own scale sub-model:

$$\ln(\sigma_{u,j}) = \beta_0^{(s_u)} + \beta_1^{(s_u)} z_{1j} + \cdots + \beta_q^{(s_u)} z_{qj}, \qquad \text{(between-study scale sub-model)} \tag{22}$$

$$\ln(\sigma_{e,i}) = \beta_0^{(s_e)} + \beta_1^{(s_e)} w_{1i} + \cdots + \beta_r^{(s_e)} w_{ri}, \qquad \text{(within-study scale sub-model)} \tag{23}$$

where the moderators in the three sub-models need not be the same (note that the between and within-study moderators can often be different). The location coefficients, $\beta^{(l)}$, describe how moderators shift the expected effect size; $\beta^{(s_u)}$ describes how moderators change heterogeneity among studies; and $\beta^{(s_e)}$ describes how moderators change residual heterogeneity among effect sizes within studies.

The scale coefficients are additive effects on the natural logarithm of an SD. For a binary plant-versus-animal moderator, with animal-based fertiliser as the reference category, the corresponding contrasts on the natural SD scale are:

$$R_u = \frac{\sigma_{u,P}}{\sigma_{u,A}} = \exp\left(\beta_{\texttt{plant-animal}}^{(s_u)}\right), \qquad R_e = \frac{\sigma_{e,P}}{\sigma_{e,A}} = \exp\left(\beta_{\texttt{plant-animal}}^{(s_e)}\right).$$

These SD ratios directly express the multiplicative difference in heterogeneity: a ratio of 1 indicates equal SDs, and a ratio above 1 indicates greater heterogeneity under plant-based fertiliser. When software models $\ln(\sigma^2)$ rather than $\ln(\sigma)$, the corresponding SD ratio is $\exp(\beta/2)$.

This extension provides a more refined view of generalisability and transferability. Two contexts may have

© 2026. Published by The Company of Biologists under the terms of the Creative Commons Attribution License (https://creativecommons.org/licenses/by/4.0/).

similar expected effects but differ in where heterogeneity arises. Greater between-study heterogeneity means that study-level effects vary more strongly around the expected effect, making that expectation less transferable to a new study. Greater within-study heterogeneity instead means that individual effect sizes are less consistent within studies, even if study-level averages remain similarly transferable. A moderator can therefore affect the location component, either scale component, or all three simultaneously.

Our organic-conventional yield example illustrates these different possibilities. For lnRR, between-study heterogeneity was greater under plant- than animal-based fertilisers (plant-to-animal SD ratio = 2.18, 95% CrI = $[1.11, 5.55]$), whereas the difference in residual heterogeneity was uncertain (SD ratio = 1.14, 95% CrI = $[0.94, 1.40]$). For lnCVR, the pattern was different: residual heterogeneity was greater under plant-based fertilisers (SD ratio = 1.67, 95% CrI = $[1.18, 2.34]$), whereas the difference in between-study heterogeneity was highly uncertain (SD ratio = 1.05, 95% CrI = $[0.10, 8.63]$). Thus, the same moderator can influence different levels of heterogeneity for different ecological outcomes.

Importantly, the extension preserved the broad finding that context dependence differed between lnRR and lnCVR, while showing that it arose at different levels. Estimating moderator effects on between-study heterogeneity nevertheless requires sufficient replication at the study level. In our example, only six studies contained both fertiliser categories, limiting information about category-specific study effects. We therefore treat this extension primarily as an illustration of how multilevel location–scale models can separate different sources of heterogeneity. Full model specifications, assumptions, sensitivity analyses, and prediction intervals for new studies are provided in the online tutorial.

2. The manuscript does not establish that the standard deviations in the underlying crop-yield dataset all measure the same kind of variation. For example, it is not clear whether they consistently represent variation across experimental replicates, variation across years, etc. Therefore, applying a variability effect size to these standard deviations does not automatically produce a measure of temporal yield stability. In my opinion, the safer interpretation would be that the analysis measures within-group yield variability. If the authors want to make claims specifically about temporal stability, they should demonstrate that the standard deviations used in the analysis consistently represent variation through time. Otherwise, the wording should probably be updated.

[R3] We agree. The available summary data do not establish that all reported SDs represent temporal variation; they may instead reflect variation among replicates, years, or other sampling units. In this revision, we have revised the interpretation of the crop-yield analyses throughout the manuscript, replacing crop-specific claims about 'yield stability' or 'reliability' with 'within-group yield variability' (or 'mean-corrected within-group yield variability' for lnCVR). This avoids assigning a temporal or reliability interpretation to the reported SDs that is not supported by the available summary data. We retain terms such as 'stability' or 'reliability' only in generic methodological examples where they are explicitly hypothetical and are not used to interpret the crop dataset. We have made corresponding changes throughout the manuscript, figures (including captions), keywords and the online tutorial.

© 2026. Published by The Company of Biologists under the terms of the Creative Commons Attribution License (https://creativecommons.org/licenses/by/4.0/).

Original wording	Replace with
yield stability	yield variability
reliability/stability (lnVR/lnCVR)	within-group variability (lnVR/lnCVR)
within-population yield variability (i.e., stability)	within-group yield variability
yield stability; lower stability	within-group yield variability; greater relative variability
within-population stability unanalysed	within-group variability unanalysed
Example: yield variability (stability)	Example: within-group yield variability
reliability and stability; targets stability	within-group yield variability; targets variability relative to mean yield
lnCVR (reliability/stability gap)	lnCVR (within-group variability contrast)
yield gap and stability	yield gap and within-group variability
stability gap/comparison	within-group variability contrast/comparison
stability or reliability in crop-specific claims	within-group variability

3. There are several small but sometimes actually important (when they affect the meaning) typesetting and grammatical issues throughout the manuscript. Please check carefully through the manuscript to ensure that these are corrected. I have tried to flag these below but I may have missed some.
[R4] Thank you for highlighting this. We have addressed all of the specific issues identified below and carefully reviewed the entire manuscript and online tutorial for additional typesetting, grammatical, and wording issues, including those that could affect interpretation.

Line-by-line comments:
* *Page 2, line 20: 'Ecological syntheses (meta-analysis)' should probably be 'Ecological syntheses (meta-analyses)'.*
[R5] Fixed.

* *Page 3, lines 48-50: The sentence beginning 'Both levels matter for ecological inference and practical application...' is difficult to follow because the explanation*

© 2026. Published by The Company of Biologists under the terms of the Creative Commons Attribution License (https://creativecommons.org/licenses/by/4.0/).

of generalisation and predictability is inserted in the middle of the sentence. I suggest restructuring this so that the definition is given separately or in parentheses.
[R6] Reviewer 1 is correct - the sentence was difficult to follow. We revised the sentence by separating the definitions of generalisability and predictability into a separate sentence.

Original:
Both levels matter for ecological inference and practical application, where generalisation and predictability, whether a phenomenon or effect is observed consistently across different conditions or only under specific conditions, often determine whether evidence is useful.

Revised [L40-43]:
Both levels matter for ecological inference and practical application, because the usefulness of evidence often depends on its generalisability and predictability. Here, generalisability and predictability refer to whether a phenomenon or effect is observed consistently across different conditions or only under specific conditions.

* *Page 3, lines 60-62: There is a grammatical problem with 'non-constant variance across a continuous variable or among groups are called "heteroscedasticity"'. 'Variance' is singular, so this could instead read 'non-constant variance ... is termed heteroscedasticity'.*
[R7] Addressed.

* *Page 4, line 84: 'within population yield variability' should be hyphenated as 'within-population yield variability', consistent with its use elsewhere in the manuscript.*
[R8] Addressed.

* *Page 6, line 101: The Figure 1 caption states that the pooled mean effect is 'Model 2'. This should be Model 1, as Model 1 is the lnRR mean-effect analysis throughout the rest of the manuscript.* [R9] Thank you for finding this - revised.

* *Page 6, line 102: Throughout the manuscript, I suggest changing 'log variance ratio' to 'log variability ratio' for lnVR. As defined here, lnVR compares standard deviations rather than variances, so 'log variance ratio' is potentially misleading.*
[R10] We have changed "log variance ratio" to "log variability ratio" throughout the manuscript to make clear that lnVR compares standard deviations.

* *Page 7, line 140: 'Using the notation adopted in the past work Nakagawa and Santos (2012)' is awkwardly phrased (it sounds like it is missing the word 'of'). I suggest something simpler, such as 'Following the notation used by Nakagawa and Santos (2012)...'.*
[R11] Fixed.

* *Page 8, line 168: 'We used a part of the organic versus conventional crop-yield database' would read more naturally as 'We used a subset of the organic versus conventional crop-yield database'.*
[R12] Changed.

* *Page 8, line 173: 'three level meta-analysis' should be 'three-level meta-analysis'.*
[R13] Addressed.

* *Page 9, line 175: 'our model is for an illustrative purposes' should be 'our model is for illustrative purposes'.*
[R14] Deleted "an" from the text.

* *Page 9, lines 179-181: The reported I^2 values are presented as '98.11', '46.09', and '52.02', whereas I^2 is normally expressed as a percentage and Figure 2 reports the total I^2 this way. I suggest adding percentage signs here and checking that I^2 values are formatted consistently throughout the whole manuscript.*
[R15] Addressed two parts. [L169 and L219]

* *Page 12, line 218: 'The question changes focus from mean to focus on within-*

© 2026. Published by The Company of Biologists under the terms of the Creative Commons Attribution License (https://creativecommons.org/licenses/by/4.0/).

population variability' repeats 'focus' and is a bit awkward. Maybe change to 'The question shifts from mean effects to within-population variability, but the meta-analytic model remains the same.'
[R16] We have made the changes as Reviewer 1 suggested. [L205]

* *Page 12, lines 219-221: 'It would be worth mentioning that...' reads like an internal drafting note rather than final manuscript text?*
[R17] Thank you. We agree that this wording reads like an internal drafting note and have revised the sentence to state the limitation directly.

* *Page 15, lines 269-272: 'It is noted that Nakagawa et al. (2025a) introduces...' is a bit awkward. I suggest 'Nakagawa et al. (2025a) introduce more complex location-scale models...'.*
[R18] Addressed. [L261-264]

* *Page 21, line 357: Should be 'SN thanks Elena Gazzea'.*
[R19] Addressed.

* *Please check the capitalisation and punctuation of all CRediT contribution terms here for consistency, particularly 'Funding Acquisition', 'Project administration', and 'Writing - Original Draft'.*
[R20] We have revised the Author Contributions section to use consistent CRediT terminology, capitalisation, and punctuation throughout.

Reviewer 2:

Thank you for the opportunity to read this interesting work - it has changed my planned approach to an upcoming meta-analysis! Having read the work, I can confirm that it has proper controls and uses appropriate methods and tools. Appropriate statistical tests are used, and as noted by the authors, they do not claim that the modelling approach they use is the 'best' approach for their modelled dataset, rather that they illustrate a meta-analytic workflow. The sample size of the dataset they use to demonstrate this is satisfactory, and they use a dataset which has already been published elsewhere. In conduction with the linked online tutorial, the manuscript does provide sufficient detail for reproducibility, though I do have a number of comments on the tutorial (see below) to ensure it is easy to follow. The authors conclusions are supported by the data, and I cannot detect any flaws in the approach taken, though some additional information and small edits are suggested below.
[R21] We thank the reviewer for their very positive comments on our manuscript. We are particularly pleased that they found the workflow useful for their own meta-analytic work, and we greatly appreciate their thoughtful suggestions for improving both the manuscript and the online tutorial.

The only major addition I would suggest making to improve the scholarship of the manuscript is to add more information / discussion about whether this approach can be applied to meta-analyses using other measures. Standardised mean difference is briefly referred to, but this could be expanded and made more explicit. It would also be good to explicitly state whether this approach can or cannot be applied to other measures, such as odds rations or associations (e.g. r or Fisher's z), and then discuss the implications of this. Other than this, I only have a few small suggestions, detailed below.
[R22] We have expanded the scope of the discussion to distinguish the multilevel location/heterogeneity model from the complete mean-versus-variability workflow. Mean-effect measures such as the standardised mean difference, odds ratio, and Fisher's z-transformed correlation can be analysed using multilevel meta-regression, including models in which residual or study-level heterogeneity varies with moderators where the data support such models. However, these effect measures do not by themselves provide a direct contrast in within-group variability. The complete workflow requires an additional dispersion-focused effect size, such as lnVR or lnCVR, when suitable group-level summary data are available. We

© 2026. Published by The Company of Biologists under the terms of the Creative Commons Attribution License (https://creativecommons.org/licenses/by/4.0/).

also clarified the special role of standard deviations in the standardised mean difference. In this case, within-group SDs are used to standardise the difference in means, rather than being analysed directly as the outcome of interest. We added the following text:

Added [L108-121]:
We do not focus on the standardised mean difference (d) in this article because ratio-scale outcomes are common in ecology, making lnRR a natural scale. In d, within-group standard deviations are pooled and used to standardise the difference in means. Variation therefore enters primarily as a scaling quantity for the mean effect, rather than as an outcome whose difference between groups is estimated directly. Thus, although differences in within-group variability can influence the value of d, d does not directly quantify whether variability itself differs between groups. Our broader message here is to treat differences in variation themselves as targets of synthesis. Importantly, the full workflow is most direct for two-group studies reporting means and standard deviations, because a mean-effect measure such as lnRR or the standardised mean difference can be paired with lnVR or lnCVR. Standardised mean differences use pooled within-group variation to scale a mean contrast, but do not themselves estimate a between-group variability contrast. Odds ratios and correlations, commonly transformed to Fisher's z, can be fitted in multilevel location and heterogeneity models, including models in which residual or study-level heterogeneity varies with moderators. However, these measures alone do not provide the same mean-versus-variability decomposition without a separate dispersion-focused effect size.

Line 47: I would suggest changing 'studies' to 'contexts' as it is really the differences between contexts which would be of interest to the ecologist, rather than the study per se.
[R23] Addressed.

Line 47-50: It may be as this is a long sentence, but I don't find it's meaning clear. Perhaps split into two sentences, or with 'whether a phenomenon or effect is observed consistently across different conditions or only under specific conditions,' in paratheses?
[R24] Thank you. We agree that the sentence was difficult to read. As also noted by Reviewer 1 [R6], we have revised this passage by separating the definition of generalisability and predictability into a separate sentence.

Original:
Both levels matter for ecological inference and practical application, where generalisation and predictability, whether a phenomenon or effect is observed consistently across different conditions or only under specific conditions, often determine whether evidence is useful.

Revised [L40-43]:
Both levels matter for ecological inference and practical application, because the usefulness of evidence often depends on its generalisability and predictability. Here, generalisability and predictability refer to whether a phenomenon or effect is observed consistently across different conditions or only under specific conditions.

Line 64: I would change this to 'effect sizes and variance'. Because the rest of the paragraph seems to be discussion shortcomings in the treatment of variance, and the proposed framework focuses on an approach which can assess both.
[R25] Thank you for this suggestion. We have revised the sentence to explicitly refer to both effect sizes and their variability across contexts.

Original:
What is often more important is understanding when and why effect sizes vary between studies.

Revised [L57-58]:
What is often more important is understanding how both effect sizes and their variability differ across contexts.

© 2026. Published by The Company of Biologists under the terms of the Creative Commons Attribution License (https://creativecommons.org/licenses/by/4.0/).

Line 71-73: Add (Nakagawa et al., 2015; Senior et al., 2020) to support this statement. [R26] We have added Nakagawa et al. (2015) and Senior et al. (2020) to support this statement.

I really like Figure 1, it does a great job of graphically demonstrating the main points of the introductory paragraph. Both E and G are referred to as homoscedastic heterogeneity, and both F and H as heteroscedastic heterogeneity. It would be easier to talk about these differences if there were different terms to describe each of these graphs.
[R27] We are glad to hear that, and thank you for this helpful suggestion. We agree that using the same terms for panels E and G, and likewise for panels F and H, made the four scenarios difficult to distinguish. We have revised the labels in Figure 1 to explicitly distinguish the four combinations of location and scale differences: "No location or scale difference" (E), "Scale difference only" (F), "Location difference only" (G), and "Location and scale differences" (H). We have also revised the figure caption accordingly.

Original:
(E) Homoscedastic heterogeneity: mean effect and heterogeneity are the same for A and B. (F) Heteroscedastic heterogeneity: mean effects are the same, but heterogeneity differs between A and B. (G) Homoscedastic heterogeneity: mean effects differ between A and B, but heterogeneity is the same. (H) Heteroscedastic heterogeneity: both mean effects and heterogeneity differ between A and B.

Revised - Figure 1 caption:
(E) No difference in location or scale: the mean effect and heterogeneity are the same for A and B (homoscedastic). (F) Difference in scale only: mean effects are the same, but heterogeneity differs between A and B (heteroscedastic). (G) Difference in location only: mean effects differ between A and B, but heterogeneity is the same (homoscedastic). (H) Differences in location and scale: both mean effects and heterogeneity differ between A and B (heteroscedastic).

NOTE: We have removed unpublished data that had been provided for the referees in confidence.

Section 2 is very nicely described. One possible addition is on / around lines 129-130. As this paper may be read by those who are less familiar with all aspects of meta-analysis, it would be helpful to explicitly state why analysis using standardised mean difference (d) treats variation as a background assumption.
[R28] Thank you for this helpful suggestion. We have expanded these sentences to explain why variation plays a different role in the standardised mean difference. Specifically, we have clarified that within-group standard deviations are pooled and used to standardise the difference in means, so variation enters primarily as a scaling quantity (not an outcome whose difference between groups is estimated directly).

Original:
We do not focus on the standardised mean difference (d) in this article because ratio-scale outcomes are common in ecology (making lnRR a natural scale), and because the broader message of this paper is about treating differences in variation as targets of synthesis rather than as background assumptions

Revised [L108-114]:
We do not focus on the standardised mean difference (d) in this article because ratio-scale outcomes are common in ecology (making lnRR a natural scale). In d, within-group standard deviations are pooled and used to standardise the difference in means. Variation therefore enters primarily as a scaling quantity for the mean effect, rather than as an outcome whose difference between groups is estimated directly. Thus, although differences in within-group variability can influence the value of d, d does not directly quantify whether variability itself differs between groups. Our broader message here is to treat differences in variation themselves as targets of synthesis

© 2026. Published by The Company of Biologists under the terms of the Creative Commons Attribution License (https://creativecommons.org/licenses/by/4.0/).

Legend for figure 2. I would move the text 'where negative lnRR implies lower organic yield and positive lnCVR implies higher relative variability (lower stability) under organic management' much earlier in the figure legend, perhaps in the 'title' of the legend.
[R29] We have moved the explanation of the direction of lnRR and lnCVR to the beginning of the Figure 2 legend so that readers can interpret the panels more readily.

Original:
\caption{\textbf{Location-scale meta-regression showing how a moderator shifts expected effects and their predictability.} Results are shown for lnRR (A,C,E) and lnCVR (B,D,F) when organic fertiliser type (animal- vs plant-derived) is used as a categorical moderator. k denotes the number of effect sizes (number of studies in parentheses). (A,B) Orchard plots stratified by fertiliser type. Points are individual effect sizes (bubble size $\propto 1/\mathrm{SE}$; horizontal jitter for visibility). Black squares and thick horizontal bars show the fitted subgroup mean and its 95\% interval; thin whiskers show the 95\% prediction interval. The vertical dashed line indicates no difference between organic and conventional farming. (C,D) Posterior distributions for fixed effects from a Bayesian location-scale model: location (mean) parameters ($b_0^{(l)}$ and $b_\mathrm{contrast(animal-plant)}^{(l)}$) and scale (heterogeneity) parameters ($b_0^{(s)}$ and $b_\mathrm{contrast(animal-plant)}^{(s)}$), where the scale coefficients act on $\log(\sigma)$ (positive values indicate greater residual heterogeneity). The intercept corresponds to animal-based fertiliser; the contrast gives the shift for plant-based fertiliser. (E,F) Posterior distributions for the between-study (random-effect) standard deviation. Points are posterior medians; thick and thin intervals denote 66\% and 95\% credible intervals, respectively. The \texttt{R} packages: \texttt{ggplot2} \citep{wickham2011ggplot2}, \texttt{tidybayes} \citep{kay2020tidybayes} and \texttt{orchaRd} \citep{nakagawa_orchard_2023} were used to draw these plots.

Revised - Figure 2 caption:
\caption{\textbf{\rev{Location-scale meta-regression showing how a moderator shifts expected effects and their residual heterogeneity.}} Results are shown for lnRR (A,C,E) and lnCVR (B,D,F) when organic fertiliser type (animal- vs plant-derived) is used as a categorical moderator. k denotes the number of effect sizes (number of studies in parentheses). (A,B) Orchard plots stratified by fertiliser type. Points are individual effect sizes (bubble size $\propto 1/\mathrm{SE}$; horizontal jitter for visibility). Black squares and thick horizontal bars show the fitted subgroup mean and its 95\% interval; thin whiskers show the 95\% prediction interval. The vertical dashed line indicates no difference between organic and conventional farming. (C,D) Posterior distributions for fixed effects from a Bayesian location-scale model: location (mean) parameters ($b_0^{(l)}$ and $b_\mathrm{contrast(plant-\rev{animal})}^{(l)}$) and scale (heterogeneity) parameters ($b_0^{(s)}$ and $b_\mathrm{contrast(plant-\rev{animal})}^{(s)}$), where the scale coefficients act on $\log(\sigma)$ (positive values indicate greater residual heterogeneity).
The intercept corresponds to animal-based fertiliser; the contrast gives the shift for plant-based fertiliser. (E,F) Posterior distributions for the between-study (random-effect) standard deviation. Points are posterior medians; thick and thin intervals denote 66\% and 95\% credible intervals, respectively. The \texttt{R} packages: \texttt{ggplot2} \citep{wickham2011ggplot2}, \texttt{tidybayes} \citep{kay2020tidybayes} and \texttt{orchaRd} \citep{nakagawa_orchard_2023} were used to draw these plots.

Section 3 is also very nicely described and easy to follow. I have just a couple of small suggestions to ease readability. On line 191: perhaps 'a dataset' rather than 'the same dataset', as you are describing a single use of a dataset in this sentence.
[R30] We have changed “the same dataset” to “a dataset” as suggested.

On line 9 in box 1: As the text before and after the comma are complete, related thoughts, replace the comma with semi-colon, to read: '…within groups; whether outcomes…'
[R31] Thank you for pointing this out. We have revised the punctuation to more clearly separate the two related parts of the sentence, using a colon to introduce the clarification:

“...changes dispersion within groups: whether outcomes become more homogeneous or more heterogeneous.”

© 2026. Published by The Company of Biologists under the terms of the Creative Commons Attribution License (https://creativecommons.org/licenses/by/4.0/).

Lines 290-299: The model results (tutorial and figure 3) seems to suggest that organic systems using animal-based fertilizers are less variable (more predictable) than conventional farming - the estimate is -1.3. However, this is not mentioned in either the manuscript or the tutorial. I appreciate that the focus of the manuscript is the method rather than the specific results, but this seems like an omission and the interpretation of this number should be made explicit. Alternately, if I have misunderstood the interpretation of this -1.3, this would suggest that greater explanation for the reader is needed.

[R32] The reviewer is correct that the scale intercept of approximately -1.3 was not explicitly interpreted in the manuscript or tutorial, and we agree that this could lead to confusion. This parameter does not represent lower yield variability in organic than in conventional farming. Since Model 3 uses lnRR as the response, it represents the scale intercept for the animal-based reference category on the log-σ scale and indicates the residual standard deviation of lnRR effect sizes around their expected value. Specifically, β0(s)=−1.30 corresponds to σ = exp(−1.30) ≈ 0.27. We have now clarified this interpretation in both the manuscript and the online tutorial, and explicitly distinguished the scale intercept from a direct organic-conventional comparison of yield variability.

Original:
The scale part (modelled on the log-σ scale) indicated that the plant-based system tended to be more variable (less predictable)($\beta^{(s)}_{\texttt{animal-plant}}$ = 0.17, 95% CrI = [-0.02, 0.37]), corresponding to about a 19% increase in residual heterogeneity for plant-based fertilisers (multiplicative factor exp(0. 17) ≈ 1. 19), with credible overlap around no difference.

Revised [L288-295]:
The scale intercept for the animal-based reference category was $\beta_0^{(s)}$ = − 1. 30 (95\% CrI = 0 [− 1. 45, − 1. 15]), corresponding to a residual standard deviation of σ = exp(− 1. 30) ≈ 0. 27 on the lnRR effect-size scale. This parameter describes residual heterogeneity in lnRR effect sizes around their expected value for animal-based systems; it is not a direct comparison of yield variability between organic and conventional farming. The scale contrast suggested a tendency towards greater residual heterogeneity under plant-based fertilisers ($\beta_{\rev{\texttt{plant-animal}}}^{(s)} = 0.17$, 95% CrI = [− 0. 01, 0. 35]), corresponding to about a 19% increase in residual heterogeneity relative to animal-based fertilisers (exp(0. 17) ≈ 1. 19), although the credible interval overlapped zero (Fig. 3A,C,E; see also Box 3).

In figure 3 A and B, I would suggest switching the order of 'plant' and 'animal', so that 'animal' is first and the order matches that in graphs C and D (of 'intercept' then 'contrast'). In C and D, I would suggest switching the order of l and s (currently s then l, change to having l at the top of the graph) so it matches the order in both the text and the figure legend.

[R33] We have revised Figure 3 so that animal-based fertiliser is shown first in panels A and B, consistent with its role as the reference level and intercept in panels C and D. We have also reordered the parameters in panels C and D so that the location parameters are shown above the scale parameters, matching the order used in the manuscript text and figure legend. These changes affect only the plotting order; the factor reference level, model specification, and model estimates were unchanged.

NOTE: We have removed unpublished data that had been provided for the referees in confidence.

Line 315: I think this should be metafor and glmmTMB (as the models presented were done in brms), though I would argue that the models in metafor are not comparable, so I would suggest rewording to: 'for comparable models using glmmTMB, see the online tutorial'.

[R34] Revised the sentence as suggested.

Online tutorial

© 2026. Published by The Company of Biologists under the terms of the Creative Commons Attribution License (https://creativecommons.org/licenses/by/4.0/).

1) Add a direct link to the dataset, and explicitly instruct the user to download this and load it so the code can be used unmodified.
[R35] We have added a direct download link to the dataset and explicit instructions for downloading the file, placing it in the data/ directory, and loading it using here::here(). This allows the tutorial code to be run without modification. Here is the updated online tutorial: https://ayumi-495.github.io/one-dataset-four-meta-analyses/.

2) In model 1, brms, meta-analysis (overall effect), a paragraph is repeated twice: 'One important point concerns how random effects are reported. In brms, random-effect estimates are presented as standard deviations (SD), whereas metafor reports variances. Therefore, to compare estimates between the two packages, the standard deviations reported by brms must be squared to obtain the corresponding variance estimates.'
[R36] Thank you for noting this duplication. We have removed the repeated paragraph so that the explanation of the difference between random-effect SDs reported by brms and variances reported by metafor now appears only once.

3) Use of the function brm in brms requires a version of Rtools which is compatible with the R version being used. It may be helpful to users to add a note about this, as most other parts of the code (see exception below) run without any edits / additions needed.
[R37] We have added a short note explaining that, on Windows, brm()requires an installation of Rtools that is compatible with the version of R being used.

4) In the glmmTMB section, there is a typo, in 'For meta-analysis, this grouping dactor should always have one level', correct to 'factor'.
[R38] Corrected.

5) When I run the model 2 InCVR section for both metafor and glmmTMB, I get very slightly different results to those in the tutorial e.g. for the metafor overall effect my estimate is 0.3397 and the I2_Total is 55.863504. I'm not sure what is causing this, as my results for model 1 and lnVR are identical. I also get a slightly different result to that reported in the tutorial for models mod_lnCVR_ls, mod_lnCVR_mr2, glmm_lnRR1 and glmm_lnCVR1. Model glmm_lnRR2 gives the error message: Error in fitTMB(TMBStruc) : negative log-likelihood is NaN at starting parameter values
[R39] Thank you very much for identifying these discrepancies and for providing the numerical results and error message. We investigated each of these cases in a clean R session and were able to reproduce the reviewer's reported metafor lnCVR results exactly (overall estimate = 0.3397; I2_Total =55.863504).

The small numerical differences in the lnCVR analyses were caused by a change in the default behaviour of metafor. The original tutorial was generated using metafor 4.8-0, whereas under metafor 5.0-1 the second-order Taylor bias correction for the coefficient of variation ratio is applied by default. We have made this analytical choice explicit by specifying correct = TRUE when calculating lnCVR. We recalculated the affected lnCVR analyses and updated the corresponding tutorial output, manuscript values, and figures accordingly.

These updates resulted in only minor numerical changes and did not alter the interpretation of the results. For example, the total I2 for lnCVR changed from 56.53% to 55.86%. In Model 4, the plant-animal location contrast remained 0.08, with its 95% CrI changing slightly from [-0.16, 0.32] to [-0.16, 0.31], while the scale contrast changed from 0.52 [0.18, 0.86] to 0.51 [0.17, 0.86]. The substantive conclusions were unchanged. Explicitly specifying this argument makes the bias-correction choice reproducible across metafor versions in which the default differs.

We identified a separate issue underlying the glmm_lnRR2 error.
In the original tutorial workflow, the variance-covariance object (vcv) used by the glmmTMB models could be inherited from the preceding lnVR or lnCVR analysis rather than being reconstructed for the response currently being analysed.
We reproduced the reported negative log-likelihood is NaN at starting parameter values

© 2026. Published by The Company of Biologists under the terms of the Creative Commons Attribution License (https://creativecommons.org/licenses/by/4.0/).

error when the stale lnVR vcv object was used. We have now revised each glmmTMB section to construct the response-specific vcv object within the relevant code chunk. With this correction, glmm_lnRR1, glmm_lnCVR1, and glmm_lnRR2 all converged successfully with convergence code 0 and positive-definite Hessians.

These corrections did not require changes to the model formulas, random-effects structures, factor reference levels, starting values, optimisers, or data inclusion criteria. We have updated the tutorial and regenerated the affected outputs and figures.

6) In model 3-4 section 'It is best to treat the metafor models presented above as approximations' should read 'below'.
[R40] Corrected.

Second decision letter

MS ID#: bio.062872R1

MS Title: One dataset, four meta-analyses: synthesising mean effects and heterogeneity within and among populations

Authors: Haley Lacza; Ayumi Mizuno; Coralie Williams; Malgorzata Lagisz and Shinichi Nakagawa

I am happy to tell you that your manuscript has been accepted for publication in Biology Open, pending our standard publication integrity checks. It was accepted on 1st September 2026.

© 2026. Published by The Company of Biologists under the terms of the Creative Commons Attribution License (https://creativecommons.org/licenses/by/4.0/).
